# Evolutionary plasticity of restorer-of-fertility-like proteins in rice

**DOI:** 10.1038/srep35152

**Published:** 2016-10-24

**Authors:** Joanna Melonek, James D. Stone, Ian Small

**Affiliations:** 1 ARC Centre of Excellence in Plant Energy Biology, The University of Western Australia, 6009 Crawley, Western Australia; 2 Institute of Botany, Czech Academy of Sciences, Zámek 1, Průhonice, 25243 Czech Republic

## Abstract

Hybrid seed production in rice relies on cytoplasmic male sterility (CMS) induced by specific mitochondrial proteins, whose deleterious effects are suppressed by nuclear *Restorer of Fertility* (*RF*) genes. The majority of RF proteins belong to a specific clade of the RNA-binding pentatricopeptide repeat protein family. We have characterised ‘*restorer-of-fertility-like’ (RFL)* sequences from 13 *Oryza* genomes and the *Brachypodium distachyon* genome. The majority of the *RFL* sequences are found in genomic clusters located at two or three chromosomal loci with only a minor proportion being present as isolated genes. The *RFL* genomic cluster located on *Oryza* chromosome 10, the location of almost all known active rice *RF* genes, shows extreme variation in structure and gene content between species. We show evidence for homologous recombination events as an efficient mechanism for generating the huge repertoire of RNA sequence recognition motifs within RFL proteins and a major driver of RFL sequence evolution. The RFL sequences identified here will improve our understanding of the molecular basis of CMS and fertility restoration in plants and will accelerate the development of new breeding strategies.

Proper functioning of a plant cell depends on coordinated expression of genes encoded in all three genomes (nuclear, mitochondrial, plastid). Despite this functional interdependence, inheritance and evolution of the nuclear and organellar genomes are quite different. Whereas nuclear genes are regularly re-shuffled during meiosis, the organellar genes are typically strictly uniparentally inherited^1^. Organellar genes and the nuclear genes encoding organellar proteins co-evolve via compensatory mutations^1^,^2^. The tight epistasis between nuclear and organellar genotypes can be the source of genetic incompatibilities after a new nuclear genome has been introduced into a cytoplasmic background^1^. Mitochondrial-nuclear genome incompatibilities caused by mitochondrially-encoded traits can lead to plant sterility and in this way can prevent interspecific crosses by creating barriers between the nucleus and foreign mitochondria^1^,^2^,^3^. Cytoplasmic male sterility (CMS) is one of the best investigated examples of mitochondrial-nuclear genome incompatibility as it has a direct application in production of F1 hybrids in crops^4^,^5^.

In breeding hybrids, CMS is used as a way of controlling self-pollination of autogamous plants^4^,^5^. The mitochondrial genomes of CMS plants encode proteins that induce the abortion of pollen development and thus male sterility^4^,^6^. The majority of investigated CMS-associated ORFs feature chimeric structures composed partly of conserved mitochondrial gene sequences (often the 5′ region containing transcription and translation starts) and partly of unique sequences^6^,^7^,^8^. How CMS-associated ORFs lead to the abortion of pollen development is largely unknown^4^,^9^. Deficiencies in mitochondrial energy production, toxicity of the CMS-causing proteins and premature programmed cell death of tapetal cells have been proposed as possible scenarios explaining this phenomenon^4^,^6^,^10^. In nature and in F1 hybrid breeding programs, expression of these mitochondrial CMS-inducing ORFs is controlled by restorer of fertility (RF) proteins. Nuclearly encoded RF proteins are imported into mitochondria where they block the expression of CMS-inducing ORFs^8^. The exact mechanism by which they achieve this is not known, but it is presumed that RF proteins bind directly to the CMS-inducing transcript, preventing translation or inducing RNA cleavage^4^,^11^. Recent studies indicate that additional proteins may be required for proper function of RF proteins in rice^12^,^13^ and restorer-of-fertility-like (RFL) proteins in *Arabidopsis thaliana*^14^,^15^.

RFL proteins form a distinct group of pentatricopeptide repeat (PPR) proteins^11^,^16^, a huge family of sequence-specific organellar RNA-binding proteins that participate in a wide range of post-transcriptional processes leading to the maturation of organellar transcripts^17^,^18^. PPR proteins can be divided into P and PLS subfamilies^17^,^18^,^19^. PLS-class proteins are predominantly involved in RNA editing, whereas P-class PPR proteins are involved in stabilisation of organellar transcripts and intron splicing^17^,^18^,^19^. RFL proteins belong to the P-class PPR subfamily and are characterised by the presence of tandem arrays of 15 to 20 PPR motifs each composed of 35 amino acid residues^16^. High substitution rates observed for particular amino acids within otherwise very conserved PPR motifs, indicating diversifying selection, prompted the conclusion that these residues might be directly involved in binding to RNA targets^16^. These discoveries provided the foundation for the development of a “PPR code” which allows the prediction of RNA targets of naturally occurring PPR proteins^20^,^21^,^22^ as well as the design of synthetic PPR proteins that can bind RNA molecules of interest^23^,^24^. Sequence specificity is ensured by distinct patterns of hydrogen bonding between each RNA base and the amino acid side chains at positions 5 and 35 in the aligned PPR motif 25.

In recent years, genes encoding RF proteins have been cloned from various plant species (reviewed in refs 5 and 11). The best studied cereal CMS/Rf systems are in the genus *Oryza* (Supplementary Table S1 and references therein). The *Rf-1* locus located on chromosome 10 in rice has been isolated independently by several groups and shown to restore fertility in BT-type CMS (Supplementary Table S1)^26^,^27^,^28^. *Rf-1* encodes a protein composed of 791 amino acids comprising 18 tandem PPR motifs^26^,^27^. Later it was discovered that in the elite restorer line Minghui63 (MH63), the locus on chromosome 10 encodes two *Rf-1* genes, which were named *Rf1a* and *Rf1b*^8^. *Rf1b* orthologs in 6 restoring and 6 non-restoring lines differ by single amino acid substitutions^8^. RF1A was proposed to restore male fertility by blocking production of the suspected CMS-inducing protein ORF79 via endonucleolytic cleavage of the *B-atp6/orf79* transcript. RF1B most likely also causes degradation of this dicistronic mRNA via an unknown mechanism^8^. *RF1a* has been demonstrated to be epistatic to *RF1b*^8^.

In CMS-WA rice, an interaction involving mitochondrially encoded, CMS-conferring Wild Abortive 352 (WA352) protein, nuclearly encoded COX11 protein and two *RF* genes has been described^29^. It was proposed that WA352 protein, produced exclusively in the tapetum of CMS-WA plants, interacts with COX11 and suppresses its function^29^. This suppression induces premature programmed tapetal cell death and leads to pollen abortion^29^. Two genes, *Rf3* and *Rf4,* located on rice chromosomes 1 and 10 respectively, can restore CMS-WA^30^,^31^. PPR9-782-M and PPR782a, RF4 candidate proteins from the elite restorer line MH63 and cultivar IR24 respectively, are 86% identical to the RF1A restorer of CMS-BT rice and are encoded within the same chromosomal region^32^,^33^. Recently, two genes designated *Rf5* and *Rf6* were determined to restore fertility in Hong-Lian CMS rice^12^,^13^.

Three candidate *RF* genes and several additional *RFL* genes have been reported in sorghum^34^,^35^,^36^. The first to be identified was the *Rf1* locus on chromosome 8, which, unlike all other RF proteins so far, encodes a PLS-class PPR protein^36^. The *Rf2* gene located within a genomic cluster of *RFL* genes on chromosome 2 has been reported to restore fertility in the A1 cytoplasm^34^ and *Rf5* located on chromosome 5 restores fertility in both A1 and A2 cytoplasms^35^. The mitochondrially-encoded, CMS-associated ORFs causing sterility in sorghum A1 and A2 cytoplasms have not been identified yet.

Apart from these examples from rice and sorghum, no *RF* genes encoding PPR proteins have been cloned and characterised from other cereal crops. Although CMS-based hybrid systems can be established without *Rf* sequence information, such knowledge will certainly accelerate marker-assisted selection and transfer of *Rf* alleles into elite breeding lines through traditional breeding. The obtained sequences could, however, also be directly introduced into desired lines by transgenic approaches. Intensive efforts are being made to identify restorer genes for *msm1* and *msm2* male-sterile cytoplasms in barley, and recently high-resolution genetic and physical mapping narrowed the region containing the *Rfm1* locus in barley to the short arm of chromosome 6H^37^. Similarly, although several major restoring alleles in maize including *Rf1* for CMS-Texas (CMS-T)^38^, *Rf3* for CMS-USDA (CMS-S)^39^ and *Rf4* for CMS-Charrua (CMS-C)^40^ have been mapped for decades, their sequences remain to be isolated.

With recent advances in sequencing technology, a whole plethora of fully or partially sequenced plant genomes and transcriptomes have become available. We took advantage of these large-scale data sets to systematically identify and characterise 158 *RFL* genes from 13 rice genomes and *Brachypodium distachyon*. We have compared several alternative methods for distinguishing RFL sequences from other P-class PPR proteins, resulting in a rapid but robust and effective pipeline. Subtle but characteristic features of PPR motifs in RFL proteins separate them from the remaining P-class PPR proteins in cereals. Only a few *RFL* genes are found as singlets with the vast majority organised into genomic clusters showing relatively low interspecific synteny. To explore mechanisms underlying the high sequence diversity we analysed an *RFL* cluster in nine rice species and were able to confirm recombination events as major factors driving evolution of this unusual subfamily of PPR proteins. We discuss the possible mode of action of RFL proteins and the implications for plant fertility. The catalogue of cereal RFL sequences gathered in this study will be a useful resource for experimental approaches and will help in identifying *RFL* sequences in newly mapped genetic regions predicted to contain a restorer-of-fertility gene.

## Results

### Bioinformatics pipeline for identifying *RFL* sequences in genomic sequence data

**Figure 1:**
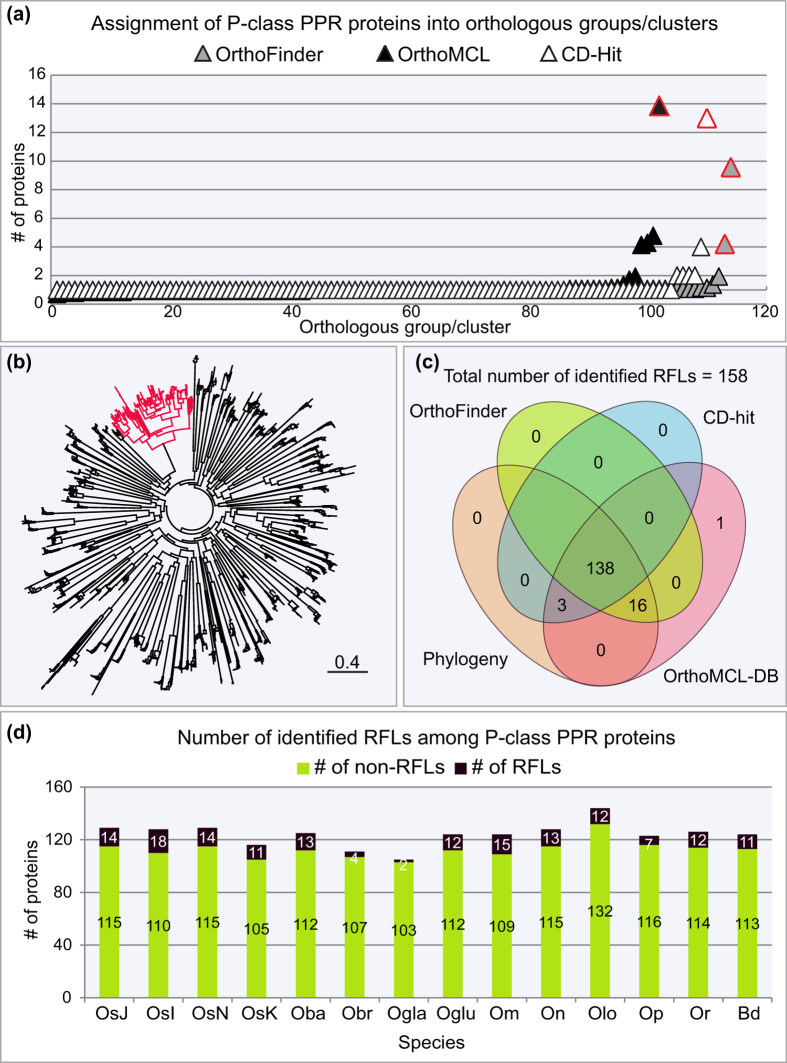
Identification of RFL sequences in 13 *Oryza* genomes and the *Brachypodium distachyon* genome. (**a**) Comparison of the assignment of the P-class PPR proteins into sequence clusters by OrthoFinder, OrthoMCL and CD-Hit. For OrthoMCL and OrthoFinder, P-class PPR sequences from all 14 genomes were analysed at once and the average number of RFL sequences per genome is shown. For CD-Hit analysis, each genome was analysed separately and only the representative outcome of clustering the sequences from *O. sativa japonica* is shown. Clusters composed of RFL sequences are highlighted in red. (**b**) Radial tree of 1736 P-class PPR-protein sequences from 13 rice genomes and the *B. distachyon* genome, as well as 36 reference RFL sequences^16^. Sequences were aligned with MAFFT v7.187^66^ and the tree constructed using FastTree 2.1.8 software^67^. The tree was visualised in Geneious 8.1.6 (www.geneious.com). The RFL clade is highlighted in red. (**c**) Comparison of the number of RFL sequences identified by the four different approaches illustrated by a Venn diagram. Out of 158 identified RFL sequences, 138 were identified by all four methods and additional 16 sequences were identified by all methods other than CD-Hit. Three sequences were not found by OrthoFinder and one sequence was identified only by OrthoMCL. **(d**) Contribution of RFL sequences to the total number of P-class PPRs in 13 *Oryza* genomes and the genome of *B. distachyon*. The total number of P-class PPR sequences per plant species is highlighted in light green and the number of RFL sequences in black, respectively.

Thirteen *Oryza* genomes and the *Brachypodium distachyon* genome were obtained from public sequence depositories (Supplementary Table S2). Introns are extremely rare or absent within *RFL* coding sequences^16^, allowing accurate annotation of *RFL* sequences from six frame translations of genomic DNA. Predicted ORFs were screened for PPR motifs using hidden Markov models^41^. Out of 9.4 million potential ORFs in the 14 genomes, 8729 contained predicted PPR motifs (Supplementary Table S3). 1736 sequences encoding 10 or more P-class PPR motifs were retained for further analysis. The number of P-class PPR proteins composed of ten or more PPR motifs varied from 105 in *Oryza glaberrima* to 144 in *Oryza longistaminata* (Supplementary Table S3). *RFL* genes generally show higher sequence similarity to their intra-specific paralogs than to putatively orthologous *RFL* genes from other species^16^. This phenomenon means that they can be identified by phylogenetic methods^16^ or other sequence clustering approaches^42^. We applied four different methods to identify the RFL proteins within the P-class PPR protein sets: creation of orthologous sequence clusters using OrthoMCL^43^ or OrthoFinder^44^, sequence clustering with CD-Hit^45^ and phylogenetic analysis following multiple alignment (Fig. 1 and Supplementary Tables S3–S5).

All four methods tested in this study converged on a set of 138 RFL sequences (Fig. 1c), with another 16 found by all methods other than CD-Hit (Fig. 1c and Supplementary Table S6). In addition, three sequences were found by all methods other than OrthoFinder and one sequence was found only by OrthoMCL (Fig. 1c and Supplementary Table S6). The preferred method depends on the scale of the analysis and the time and computational resources available. The analysed *Oryza* genomes contained from 2 (*O. glaberrima*) to 18 (*O. sativa indica*) *RFL* genes of 10 or more PPR motifs (Fig. 1d and Supplementary Table S3).

### Analysis of RFL clades in *Oryza*

**Figure 2:**
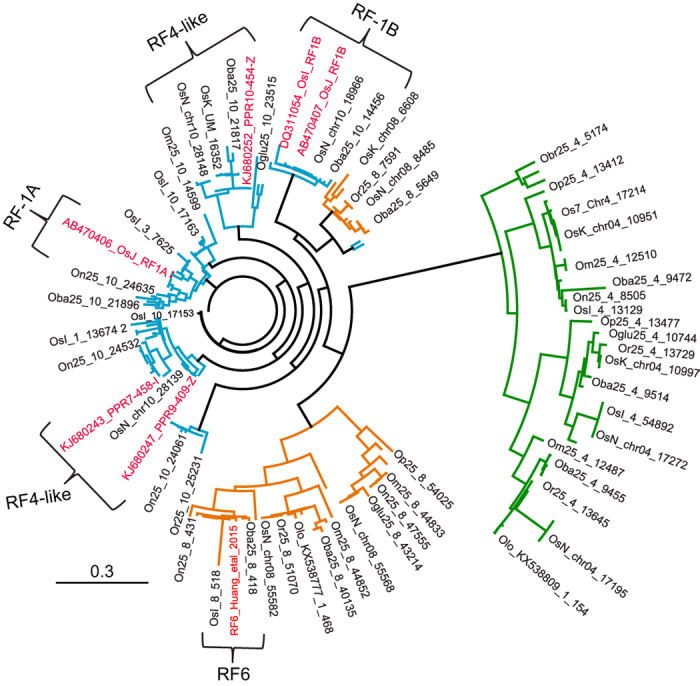
Phylogenetic analysis of RFL sequences encoded in *Oryza* genomes. Multiple sequence alignment of 147 RFL sequences identified in 13 rice genomes supplemented by 27 sequences of published RF and RFLs (Supplementary Table S1) was performed with MAFFT v7.187. The tree was generated with FastTree 2.1.5 and coloured in Geneious 8.1.6 (www.geneious.com). The *RFL* sequences located on chromosome 4 are highlighted in green, chromosome 8 in orange and chromosome 10 in blue, respectively. Published RFL sequences are highlighted in red and, apart from RF6, are all located on chromosome 10. Due to space limitations, not all tip labels are shown. The scale bar represents the number of substitutions per site.

To study the evolutionary relationships between the identified RFLs, the 147 *Oryza* RFL sequences were aligned with previously published RFs and RFLs (Supplementary Table S1) and a phylogeny constructed (Figs 1b and 2). The RFL sequences form a single monophyletic clade that stands out from other P-class clades by showing a much greater degree of recent divergence (Fig. 1b). Within the RFL clade, the sequences group into three subclades that correspond to genomic clusters located on chromosomes 4, 8 and 10, respectively (Fig. 2). The largest genomic cluster is on chromosome 10 (Fig. 2), which has been reported previously to contain active *Rf-1a, Rf-1b* and *Rf4* genes in different rice accessions^8^,^27^,^33^,^46^. The proportion of *RFL* genes in genomic clusters ranges from zero in *O. brachyantha* to 93% in *O. sativa japonica* (Table 1).

### Mechanisms contributing to the evolutionary plasticity of *RFL* genes in rice

**Figure 3:**
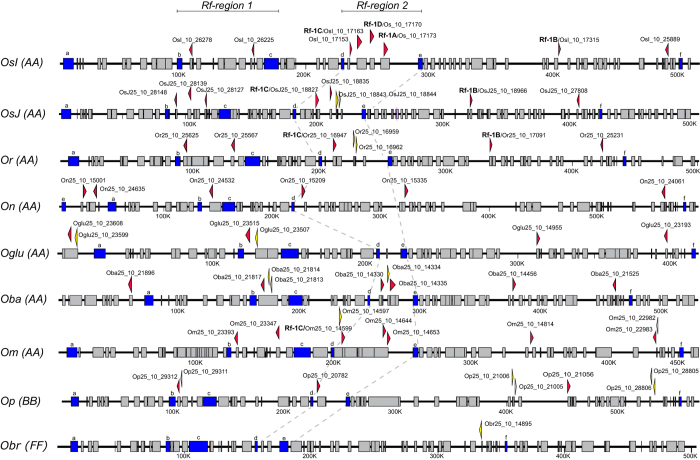
Structural analysis of the *RFL* cluster located on chromosome 10 in nine *Oryza* genomes. Genomic regions spanning ~500 kbp of chromosome 10 from *O. sativa indica* (*OsI*), *O. sativa japonica* (*OsJ*), *O. rufipogon* (*Or*), *O. nivara* (*On*), *O. glumipatula* (*Oglu*), *O. barthii* (*Oba*), *O. meridionalis* (*Om*), *O. punctata* (*Op*), and *O. brachyantha* (*Obr*), were obtained from EnsemblPlants (http://plants.ensembl.org/index.html) and gene annotations were visualized in KONG Cloning Suite (www.kongcloning.com). *Oryza* genome types (AA, FF, BB) are shown in parentheses. Genes annotated in EnsemblPlants are shown as boxes, with conserved genes listed in Supplementary Table S7 highlighted in blue. *RFL* genes are shown as arrowheads, with red indicating ten or more PPR motifs and yellow less than ten. The direction of the arrowhead indicates the orientation of the *RFL* gene. Two regions described previously to contain active *RF* genes are indicated as Rf-region 1^33^ and Rf-region 2^28^, and are located between conserved genes b-c and d-e, respectively. The annotation of the *RFL* genes (*Rf-1A-D*) is based on the phylogenetic tree presented in Supplementary Figure S3. The *Rf-1B* gene shown in the figure has been annotated according to^8^ and is different from the *Rf-1B* gene named by others^28^,^68^. The dashed lines connect syntenic loci between species. The conserved genes encode the following proteins alpha-galactosidase (**a**), KH-domain-containing protein (**b**), DNA-directed RNA polymerase (**c**), acyltransferase (**d**), serine/threonine-protein kinase (**e**), glutamyl-tRNA reductase (**f**).

Various mechanisms including homologous recombination, gene conversion, duplication and selection have been proposed to contribute to the genome-wide diversity of *RFL-*gene loci in plants^16^,^47^,^48^. In order to investigate such phenomena within the largest rice *RFL* cluster on chromosome 10, the corresponding regions spanning ~500 kbp of nine *Oryza* species were compared (Fig. 3). Local pairwise alignments revealed that the colinearity of the genomic sequences tends to break at the sites of *RFL* loci (Supplementary Figure S1). The number of *RFL* genes in the cluster composed of 10 or more PPR motifs varied from 8 in *O. sativa indica* to zero in *O. brachyantha* (Fig. 3, Supplementary Table S7). Within the cluster, two regions carrying a variable number of *RFL* genes can be distinguished (Fig. 3). The first is located between two conserved genes encoding a KH-domain protein and a DNA-directed RNA polymerase (Fig. 3). These flanking genes identify this region as that carrying the *Rf4* restorer gene in *O. sativa japonica* Zhonghua 11^33^ (Supplementary Figure S2). The second region is located between genes encoding an acetyltransferase and a serine/threonine-protein kinase (Fig. 3 and Supplementary Table S7). These flanking genes identify this region as that carrying the *RF1a* restorer^8^,^26^,^28^ (Supplementary Figure S2). Interestingly, *O. meridionalis* and *O. barthii* both contain an *RFL* gene with high sequence similarity to *Rf-1A* in *O. sativa indica* (Fig. 3). In *O. nivara* the Rf-region 2 seems to have been “broken” and subsequently translocated upstream in the cluster as indicated by the presence of two *RFL* sequences and the gene encoding the kinase protein (Fig. 3). Apart from the *RFL* genes located within the Rf-regions 1 and 2, several other genes are found nearby, including *Rf-1B*^8^ and another *RFL* gene located downstream in the cluster (Fig. 3). Both genes are present in all rice species carrying the A genome type (Fig. 3). In *O. glumipatula* and *O. barthii* a single *RFL* gene was found to be located upstream of the conserved gene encoding the alpha-galactosidase (Fig. 3 and Supplementary Table S7). It seems possible that an *RFL* gene located at this position in *O. nivara* was involved in a recombination event that has caused the partial translocation of Rf-region 2 (Fig. 3). The three *RFL* sequences found within the genomic cluster in *O. punctata* which carries the B genome type differ from the other *RFLs* found in the cluster. Two of the sequences form a distinct branch in the phylogenetic tree shown in Supplementary Figure S3, reflecting the evolutionary distance between A and B genome types in *Oryza*.

The results of the structural analysis of the *RFL* loci in all nine rice accessions and the high sequence similarity of the *RFL* genes (Fig. 3 and Supplementary Figure S3), suggest that the structural complexity of *RFL* clusters originates from gene duplications allowing for homologous recombination and unequal crossing over to take place.

Recombination analysis of *RFL* sequences located in the cluster on chromosome 10 in *O. sativa indica* with the Recombination Detection Program (RDP4)^49^ identified several potential recombination events (Supplementary Figure S4) and emphasises the chimeric structure of *RFL* genes. Such recombination events can lead to translocations and insertions of partial or whole *RFL* sequences within a cluster and by doing so will contribute to the overall sequence plasticity. Insertion of a partial *RFL* sequence within an already present *RFL* gene has the immediate consequence of altering RNA recognition by the gene product. In the longer term, duplicated *RFL* genes will diverge and slowly gain the ability to bind new mitochondrial RNA targets.

### Duplication, deletion, insertion and transposition of PPR motifs in *RFL* genes

**Figure 4:**
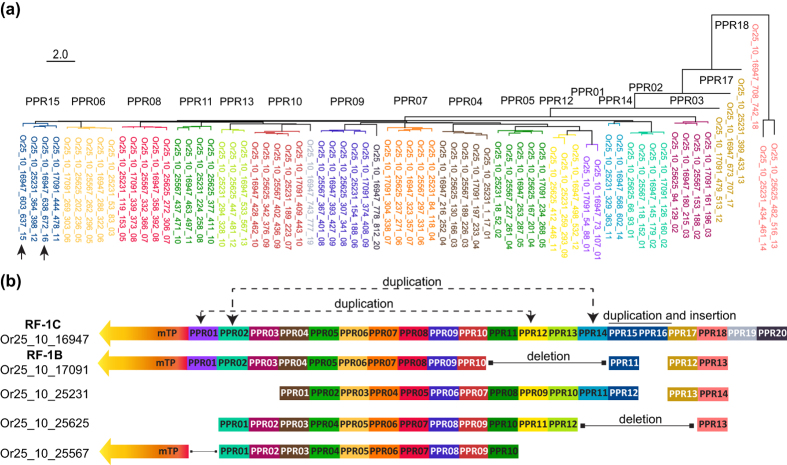
Analysis of the PPR motifs present in RFL proteins encoded within the cluster on chromosome 10 in *O. rufipogon.* (**a**) Tree illustrating the phylogenetic relationships between PPR motifs extracted from protein sequences and numbered starting from the amino-terminus. In total, 69 PPR motifs from 5 RFL sequences have been aligned. (**b**) Schematic representation of RFL proteins identified in *O. rufipogon*. The PPR motifs are coloured and numbered according to the tree in (**a**). Putative duplication, deletions and insertions of PPR motifs have been indicated.

The modular structure of PPR proteins implies that not only duplications of whole genes but also motif duplications, deletions, insertions or transpositions by recombination can give rise to functional protein variants with altered target recognition^48^. To look for evidence of such events, PPR motifs from all RFL proteins encoded on chromosome 10 in *O. rufipogon, O. sativa indica* and *O. sativa japonica* were aligned and used to build a distance tree (Fig. 4a). These three species were chosen because they are the most important with respect to the development of CMS-based breeding systems in rice. The distance tree of *O. rufipogon* PPR motifs revealed duplications and insertions, a transposition, and also deletions of one or several PPR motifs (Fig. 4b). Similar results were seen for *O. sativa indica* and *japonica* (Supplementary Figures S5 and S6).

### Characteristic features of RFL proteins

**Figure 5:**
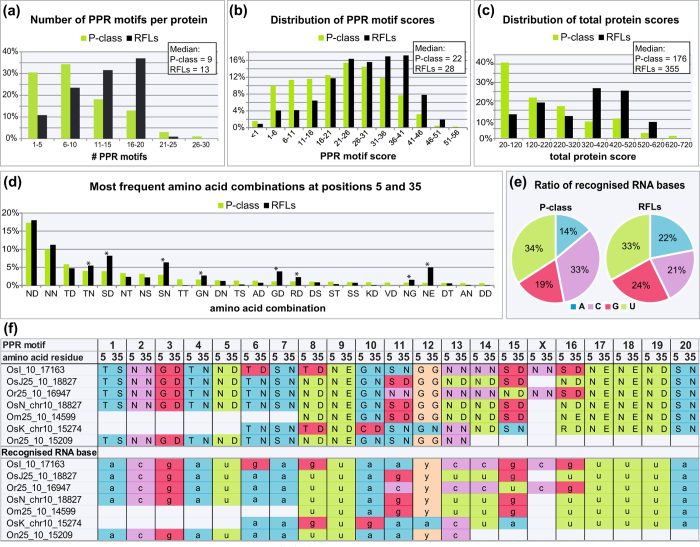
Characterisation of RFL proteins from *Oryza* and *B. distachyon*. (**a**) Number of PPR motifs in the identified P-class and RFL proteins. (**b**) Distribution of PPR motif scores for P-class and RFL proteins. The scores are domain scores per motif from hmmsearch using the P motif to search^41^. (**c**) Distribution of total protein scores (full sequence scores from hmmsearch using the P motif to search). (**d**) Frequency of amino acid combinations at positions 5 and 35 of PPR motifs in RFL and P-class PPR proteins. The 25 most frequent combinations are shown, sorted by abundance in P-class PPRs. Asterisks illustrate the combinations that are significantly more frequent in RFLs compared to P-class PPRs (p < 0.01) as listed in Supplementary Table S8. (**e**) Proportion of predicted recognised RNA bases (A, C, G, U) for P-class and RFL proteins. For the calculations, the set of combinations that add up to at least 50% of all combinations was used (Supplementary Table S9). (**f**) Combinations of amino acid residues at positions 5 and 35 of PPR motifs and the RNA sequence predicted to be recognised by these combinations^20^ for the Rf-1C orthologues^26^ located within the Rf-region 2 of the *RFL* cluster on chromosome 10.

The high diversity and rapid evolution of *RFL* sequences might suggest relaxed selection on them, but by several criteria they appear to be closer to consensus PPR proteins than other, more highly conserved members of the family. RFL proteins tend to have more PPR motifs per protein (Fig. 5a), and these motifs are generally better matches to the PPR consensus (as judged by hmmsearch scores) than those in other P-class proteins (Fig. 5b). The differences are even more prominent when total protein hmmsearch scores are compared (Fig. 5c). The sequence logos obtained from the analysis of ~34000 P-class PPR motifs and ~3000 motifs extracted from RFL proteins are almost identical (Supplementary Figure S7). The frequency with which different base-recognising combinations occur within RFL PPR motifs is generally similar to that seen for other P-class PPR proteins, but there are some subtle but significant differences (Fig. 5d). The commonest purine-recognising combinations in P-class PPR proteins are generally TD (G) and TN (A), but in RFL proteins they are SD (G) and SN (A). GD and GN are also unusually common in RFL proteins, whereas the C-recognising combinations NS and NT are unusually rare (Fig. 5d). The overall effect is that whereas P-class PPR proteins have a strong predicted bias toward pyrimidines, this is much less pronounced in RFL proteins (Fig. 5e).

High levels of sequence divergence coupled with strong conservation of overall structure are consistent with positive (diversifying) selection on specific residues within the sequence. Positive selection on the base-recognising residues within *RFL* sequences has been shown before^16^ and would be expected to lead to exceptionally high diversity at these positions. This is illustrated by a comparison of amino acid residues at positions 5 and 35 of PPR motifs extracted from *Rf-1C* orthologues^26^ in 7 rice species (Fig. 5f). High variation in these amino acid residues, supplemented by the insertion/deletion of whole PPR motifs originating from homologous recombination and unequal crossover, results in stark changes in the RNA sequence predicted to be recognized by a particular PPR protein (Fig. 5f).

## Discussion

RFL proteins share many characteristics with another protein family that is greatly expanded in plants, the family of disease resistance (R-) proteins typically composed of a nucleotide-binding site (NBS) and leucine-rich repeats (LRR)^50^. These shared characteristics include long arrays of tandem repeats within each protein and chromosomal clusters of related genes that evolve in a manner that is strikingly different to the genes around them. The rapid evolution of NBS-LRR proteins is thought to be driven by selection for pathogen recognition in an evolutionary “arms-race”^50^. The similarly rapid evolution of *RFL* genes has been proposed to be driven by an analogous “arms-race” with the plant’s own mitochondrial genome^3^,^16^. RFL proteins induce alterations in processing of their mitochondrial target RNAs, leading in some cases to complete suppression of the transcript, or at least the protein encoded by the transcript^26^,^27^,^28^,^32^,^33^. In this way they can prevent the expression of mitochondrial ORFs that otherwise lead to CMS or other forms of nuclear-mitochondrial incompatibility. The “arms-race” ensues because mitochondrial mutations causing CMS can be selected for under some circumstances (as they favour transmission of the mutated mitochondrial genome via the seed), but once CMS is frequent in a population, nuclear *RF* genes are strongly favoured in their turn. Hence the selection pressures on *R* genes and *RFL* genes are similar, probably explaining their similar evolutionary behaviour.

The high diversity in *RFL* genes is focused on the residues that contribute the most to RNA binding specificity^16^. Only one or two changes at these residues are sufficient to alter binding specificity from one target sequence to another in other PPR proteins^20^ and this is presumably true for RFL proteins too. In this case, we can confidently predict that in many cases the diverse sequences present at the same locus in different *Oryza* species (e.g. Fig. 5f) will have different functions because they can bind different RNA targets. These RNA targets are potentially predictable using the ‘code’ proposed to describe RNA sequence recognition by PPR proteins^20^,^21^,^22^. The identification of the exact binding sites of RF proteins will greatly accelerate the characterisation of the molecular mechanisms involved in fertility restoration. The mode of action of RF proteins remains largely unknown, but they are frequently implicated in endonucleolytic cleavage of their target RNAs^8^. As RF proteins are composed almost entirely of PPR motifs with no additional C- or N-terminal domains likely to possess endonuclease activity, it seems likely that additional proteins must be involved^14^,^15^.

**Figure 6:**
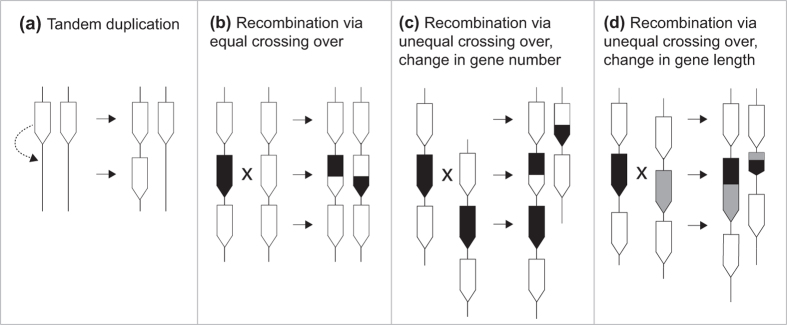
Mechanisms contributing to the evolutionary plasticity of *RFL* genes in *Oryza*. (**a**) Tandem duplication. (**b**) Recombination via equal crossing over (**c**) Recombination via unequal crossing over causing change in gene number (**d**) Recombination via unequal crossing over, causing change in gene length. Arrows represent *RFL* sequences, with differences in shading representing divergent sequences.

The data on the *Oryza* RFL clusters are consistent with recombinational processes driving most of the sequence evolution, rather than simple point mutations. The high-level of intra-cluster similarity, contrasted with the low level of inter-cluster and inter-species similarity, strongly suggests that there is sequence exchange between genes within each cluster. The most likely explanation is unequal pairing at meiosis followed by gene conversion or unequal crossing over, leading to the duplications, insertions and translocations of single PPR motifs or whole genes that we observed. A plausible depiction of some of these processes is shown in Fig. 6. The outcome is a continual flux of new *RFL* variants into the gene pool that can potentially act on any new mitochondrial sequences that appear in the equally recombinogenic mitochondrial genome. It is evident that in addition to creating functional variants, these processes will also randomly create many non-functional variants with truncations, deletions or other defects. Indeed, the *Oryza RFL* clusters analysed here are littered with presumably non-functional RFL fragments seen in the six-frame translations (some of these are shown in Fig. 3), hence the cut-off at 10 PPR motifs that we used. These fragments may however indirectly serve a purpose as inducers or generators of small non-coding RNAs as well as reservoirs of structural variation, enhancing the diversity of novel *RFL* variants.

A genome-wide analysis of the RNA-dependent RNA POLYMERASE6/DICER-LIKE4 pathway in Arabidopsis showed that several *RFL* transcripts are targeted by trans-acting short interfering RNAs (ta-siRNAs)^51^. Ta-siRNAs are produced by a mechanism that yields 21-nucleotide, phased siRNAs from *TAS* transcripts that are initially processed by miRNA-guided cleavage^51^. Similarly, rice *osa-miR1425* targets *RF1* mRNAs^52^. Out of five predicted gene targets of *miR1425* four encode RFL proteins, including *Os08g01650*, *Os10g35436*, *Os10g35640*, *Os10g35240*^52^. Plant *R* genes have been reported to be a frequent target of miRNAs^53^, especially those forming chromosomal clusters^50^. In addition, as seen for *RFL* genes in Arabidopsis, *miR2109*/*miR2118*/*miR1507* in Medicago, *miR482*/*miR2118* in tomato, and *miR6019*/*miR6020* in tobacco have been shown to guide the cleavage of transcripts of NBS-LRRs, and to trigger secondary phased siRNA production by RNA-dependent RNA polymerase^54^,^55^,^56^. The functional consequences of this potential regulation by gene-silencing pathways are still unclear.

In the past, the primary interest in *RF* genes has been their role in breeding hybrid crops, with several of them being widely used in agriculture even before their identity and mode of action were known. *RF* genes have played an important role in the development of hybrid rice varieties. The availability of suitable restorer lines was the major factor that contributed to the success of the “three-line” (CMS, maintainer and restorer line) system based on *indica* WA-CMS in hybrid rice production^57^. CMS-WA remains the most widely used single source of sterile cytoplasm for commercial hybrid seed production since 1970, not only because its pollen sterility is complete, but also because the fertility of hybrids between CMS-WA and restorer lines is readily recovered^57^. In recent years several *RF* genes have been cloned in rice (Supplementary Table S1) and with the increasing availability of sequenced genomes from other cereals, more *RF* sequences are forthcoming. Our analysis revealed about a dozen putatively functional *RFL* genes per rice species on average (Supplementary Table S3). In *O. brachyantha* and *O. glaberrima*, however, many fewer were found. In comparison to all other *Oryza* species analysed here, *O. brachyantha* has the smallest genome size (Supplementary Table S3) and far fewer gene models have been predicted in its genome sequence than in other rice species, suggesting an amplification of many gene families in domesticated rice^58^. In particular, disease resistance-related gene families (e.g. the NBS-LRR family) are highly overrepresented in *O. sativa*^58^. Whereas *O. brachyantha* branches early in the *Oryza* lineage^59^, *O. glaberrima* was domesticated from the wild progenitor *O. barthii*, an A genome species, and is related to other A genome rice species including *O. sativa japonica* and *indica*^60^. Recently, using *O. glaberrima* as a cytoplasm donor, a new *indica* CMS, designated African rice (AF)-CMS, was developed and corresponding introgression lines carrying major restorer genes from *O. glaberrima* were created^61^. The presence of active *Rf* loci in *O. glaberrima* conflicts with the low number of *RFL* genes found in this study. We suspect that the two *RFL* genes found in the available genome sequence (Table 1) probably do not represent an exhaustive catalogue of the *RFL* genes in the *O. glaberrima* genome.

In this study, we have analysed a total of 147 *Oryza* *RFL* sequences from 13 genomes. This knowledge will expedite marker-assisted selection and transfer of *Rf* alleles into elite breeding lines through traditional breeding. The identified sequences could, however, also be directly introduced into desired lines by transgenic approaches. As their fascinating properties and unusual evolutionary behaviour become better understood, other potential applications of RFL proteins besides fertility restoration are coming to the fore. PPR proteins are being investigated for their potential as custom-made RNA processing tools^24^,^62^, and of all the natural PPR proteins, RFL proteins are perhaps the best suited for biotechnological manipulation given their recurrent selection for diversity in binding site selection.

## Methods

### Genomic sequence data used in the study

The compressed fasta files containing the genomic sequence data of 13 rice genomes were either downloaded from EnsemblPlants (http://plants.ensembl.org/index.html)^63^ or the Rice Annotation Project Database (http://rapdb.dna.affrc.go.jp/gb1/index.html)^64^. The genome sequence of *B. distachyon* was downloaded from Phytozome (http://phytozome.jgi.doe.gov/pz/portal.html). A detailed list of the names of downloaded files, genome versions and release dates can be found in Supplementary Table S2.

### Bioinformatics pipeline for identifying *RFL* genes in genomic sequence data

The DNA sequences were screened for open reading frames (ORFs) in six-frame translations with the *getorf* program of the EMBOSS 6.6.0 package^65^. Predicted ORFs longer than 92 codons were screened for the presence of P- and PLS-class PPR motifs using *hmmsearch* from the HMMER 3.1b package (hmmer.org) and hidden Markov models defined by *hmmbuild*^41^. The post-processing of *hmmsearch* results was carried out according to rules described previously^41^. Sequences containing 10 or more P-class PPR motifs were retained for further analysis, as a previous study has shown that *RFL* genes are primarily comprised of tandem arrays of 15 to 20 PPR motifs^16^.

#### OrthoMCL, OrthoFinder and CD-Hit analysis

Sequence clustering methods were used to identify candidate RF sequences. One approach employed the OrthoMCL algorithm^43^ via the OrthoMCL-DB website (http://www.orthomcl.org/orthomcl/). In a second approach, OrthoFinder from https://github.com/davidemms/OrthoFinder^44^ was used to cluster P-class PPR proteins from each data set. The resulting output files were screened for groups containing reference RFLs^16^. A third approach used CD-Hit version 4.6.4 from https://github.com/weizhongli/cdhit/releases^45^. Our preliminary CD-Hit analyses on the whole pool of 1736 P-class PPR proteins showed that, even with the lowest identity threshold of 40%, CD-Hit failed to group RFLs from multiple species into a single cluster. Separately analysing the data sets from each species overcame that problem. CD-Hit was run with the settings -c 0.4 -n 2 -d 200 -G0 -aS 0.1.

#### Phylogenetic analysis

For identification of RFL sequences based on phylogeny, P-class PPR proteins were aligned with a parallel version of MAFFT v7.187^66^. The resulting alignment file was used for tree generation with FastTree 2.1.8^67^. The resulting tree was analysed in Geneious 8.1.6 (http://www.geneious.com/). The RFL clade was identified by the location of reference RFLs^16^.

#### Synteny analysis

For the analysis of genomic fragments carrying the *RFL* cluster on rice chromosome 10, the regions spanning ~500 Mbp were obtained from EnsemblPlants (http://plants.ensembl.org) and the gene annotations were visualized in KONG Cloning Suite (www.kongcloning.com). The *RFL* genes were manually annotated.

## Additional Information

**How to cite this article**: Melonek, J. *et al*. Evolutionary plasticity of restorer-of-fertility-like proteins in rice. *Sci. Rep.* **6**, 35152; doi: 10.1038/srep35152 (2016).

## Supplementary Material

Supplementary Information

## Figures and Tables

**Table 1:** Organisation of *RFLs* into clusters.

Species	Total nb of RFLs	Chromosomal location			Nb of genes in clusters	Nb of genes as singlets	% of genes in clusters^*^	Singlet(s)
		chr 4	chr 8	chr 10				
*Oryza sativa japonica*	14	3	4	7	13	1	93	OsJ25_8_8485
*Oryza sativa indica*	18	3	5	8	15	3	88	OsI_1_13674 OsI_3_7625 OsI_8_518
*Oryza sativa Nipponbare*	14	3	4	7	12	2	85	OsN_chr08_8485 OsN_chr08_431
*Oryza sativa indica, kasalath*	11**	3	4	4	8	3	73	OsK_chr08_431 OsK_chr08_6608
*Oryza barthii*	13	3	4	6	11	2	84	Oba25_8_418 Oba25_8_5649
*Oryza brachyantha*	4	2	1	0	0	4	0	Obr25_4_31256 Obr25_4_5174 Obr25_7_19230 Obr25_8_28251
*Oryza glaberrima*	2	0	2	0	0	2	0	Ogla25_8_118 Ogla25_8_6391
*Oryza glumipatula*	12	3	4	5	10	2	83	Oglu25_8_449 Oglu25_8_6078
*Oryza meridionalis*	15	4	3	8	14	1	93	Om25_10_35991
*Oryza nivara*	13	3	4	6	11	2	84	On25_8_431 On25_8_7032
*Oryza punctata*	7	2	2	3	5	2	71	Op25_8_585 Op25_8_54025
*Oryza rufipogon*	12	3	4	5	10	2	83	Or25_8_424 Or25_8_7591
Oryza longistaminata***	12	3	3	6	11	1	91	Olo_KN538908_1_31
*Brachypodium distachyon*	11	0	0	0	6 (chr.2)	5	63	Bd21_Bd1_12685 Bd21_Bd2_9844 Bd21_Bd2_49161 Bd21_Bd3_1975 Bd21_Bd4_112460

*cluster is understand as two or more genes at one chromosomal location, **location of OsK_UM_16352 within the *RFL* cluster on chromosome 10 as well as location of all *RFL* genes identified in the *O. longistaminata* genome (***) was predicted based on phylogenetic tree presented in Fig. 2.
