# Supplementary material for: Evolutionary plasticity of restorer-of-fertility-like proteins in rice
Source: Sci Rep. 2016 Oct 24;6:35152. doi: 10.1038/srep35152 (PMC5075784; doi:10.1038/srep35152)
Supplement: Supplementary Information [file srep35152-s1.pdf]

Supplementary information online

Evolutionary plasticity of restorer-of-fertility-like proteins in rice

Joanna Melonek[1*], James D. Stone[1,2], Ian Small[1]

[1] ARC Centre of Excellence in Plant Energy Biology, The University of Western Australia, 6009 Crawley, Western Australia

[2]Institute of Botany, Czech Academy of Sciences, Zámek 1, Průhonice, 25243 Czech Republic

*Corresponding author: joanna.melonek@uwa.edu.au

Supplementary Figure S1. Collinearity between 500 kbp genomic regions spanning RF-cluster extracted from chromosome 10 in rice *O. rufipogon* (*Or*), *O. sativa indica* (*OsI*) and *japonica* (*OsJ*).
Supplementary Figure S2. Arrangement of *RFL* genes in the *RF-region 1* and *2* as published by Tang *et al.*, 2014 (BACOSJNBa0041P03) and Komori *et al.*, 2004 (AB110443).
Supplementary Figure S3. Phylogenetic relationships between RFL proteins encoded by genes located within the RFL cluster located on chromosome 10 in nine *Oryza* species.
Supplementary Figure S4. Schematic sequence display showing putative recombination events between *RFL* sequences located on O. *sativa indica* chromosome 10.
Supplementary Figure S5. Analysis of the PPR motifs in the RFL proteins encoded within the cluster located on chromosome 10 in *O. sativa indica*.
Supplementary Figure S6. Analysis of the PPR motifs in the RFL proteins encoded within the cluster on chromosome 10 in *O. sativa japonica.*
Supplementary Figure S7. Sequence logos of PPR motifs from P-class PPR and RFL proteins.

Supplementary Table S1. To date published *Oryza* RF and RFL sequences.
Supplementary Table S2. Summary of data sets used in the study.
Supplementary Table S3. Identification of *RFL* sequences in genomic sequence data from 13 *Oryza* genomes and the *Brachypodium distachyon* genome.
Supplementary Table S4. Assignment of P-class PPR protein sequences into orthologous groups by OrthoMCL and OrthoFinder or clusters by CD-Hit.
Supplementary Table S5. List of RFL sequences assigned to RFL-clade by analysis with OrthoFinder, OrthoMCL-DB, CD-Hit and phylogeny.
Supplementary Table S6. Sequence overlap between the four methods used for identification of RFL proteins.
Supplementary Table S7. Genomic locations of identified *RFLs* as well as conserved non-PPR genes highlighted in the schematic drawing of *RFL* cluster on chromosome 10 in nine *Oryza* species shown in Figure 3.
Supplementary Table S8. Results of Z-score test analysis of amino acid combinations at position 5 and 35 between P-class and RFL proteins.
Supplementary Table S9. Frequency of amino acid combinations at positions 5 and 35 in P-class and RFL proteins.

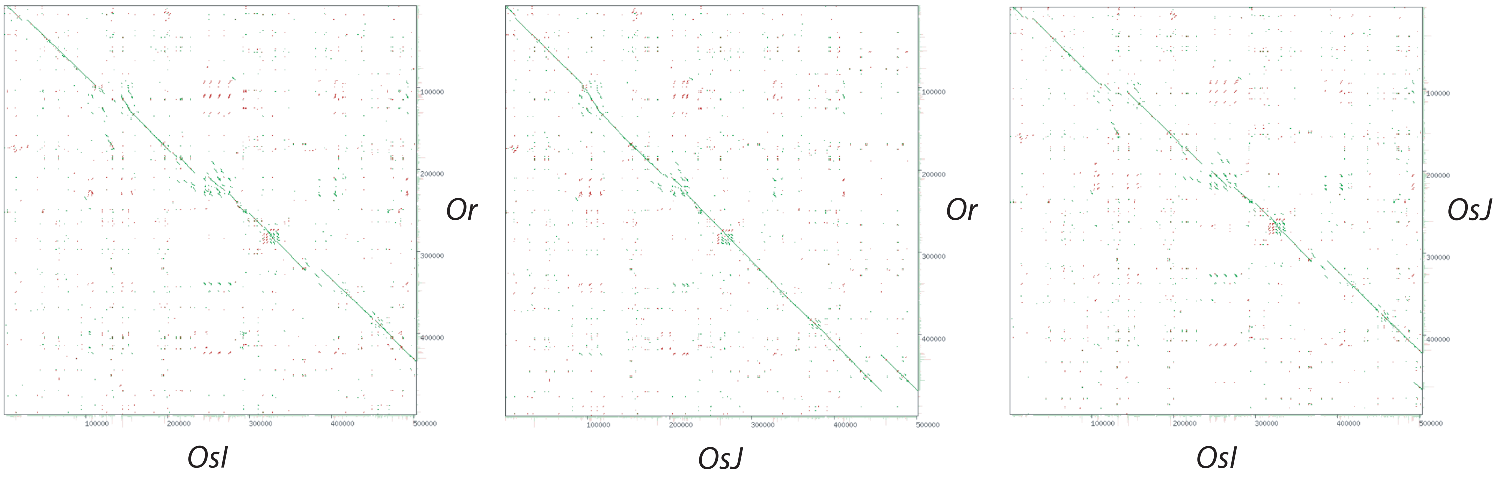

Supplementary Figure S1. Collinearity between 500 kbp genomic regions spanning RF-cluster extracted from chromosome 10 in rice *O. rufipogon* (*Or*), *O. sativa indica* (*OsI*) and *japonica* (*OsJ*). The alignment was generated with YASS (Noe and Kucherov, 2005). YASS default parameters were used except that the E-value threshold was 10^{-10} as described in Mora et al., 2010.

Mora, J. R. H., Rivals, E., Mireau, H. & Budar, F. Sequence analysis of two alleles reveals that intra-and intergenic recombination played a role in the evolution of the radish fertility restorer (Rfo). BMC Plant Biol 10, doi:Artn 3510.1186/1471-2229-10-35 (2010).

Noe, L. & Kucherov, G. YASS: enhancing the sensitivity of DNA similarity search. Nucleic Acids Res 33, W540-W543, doi:10.1093/nar/gki478 (2005).

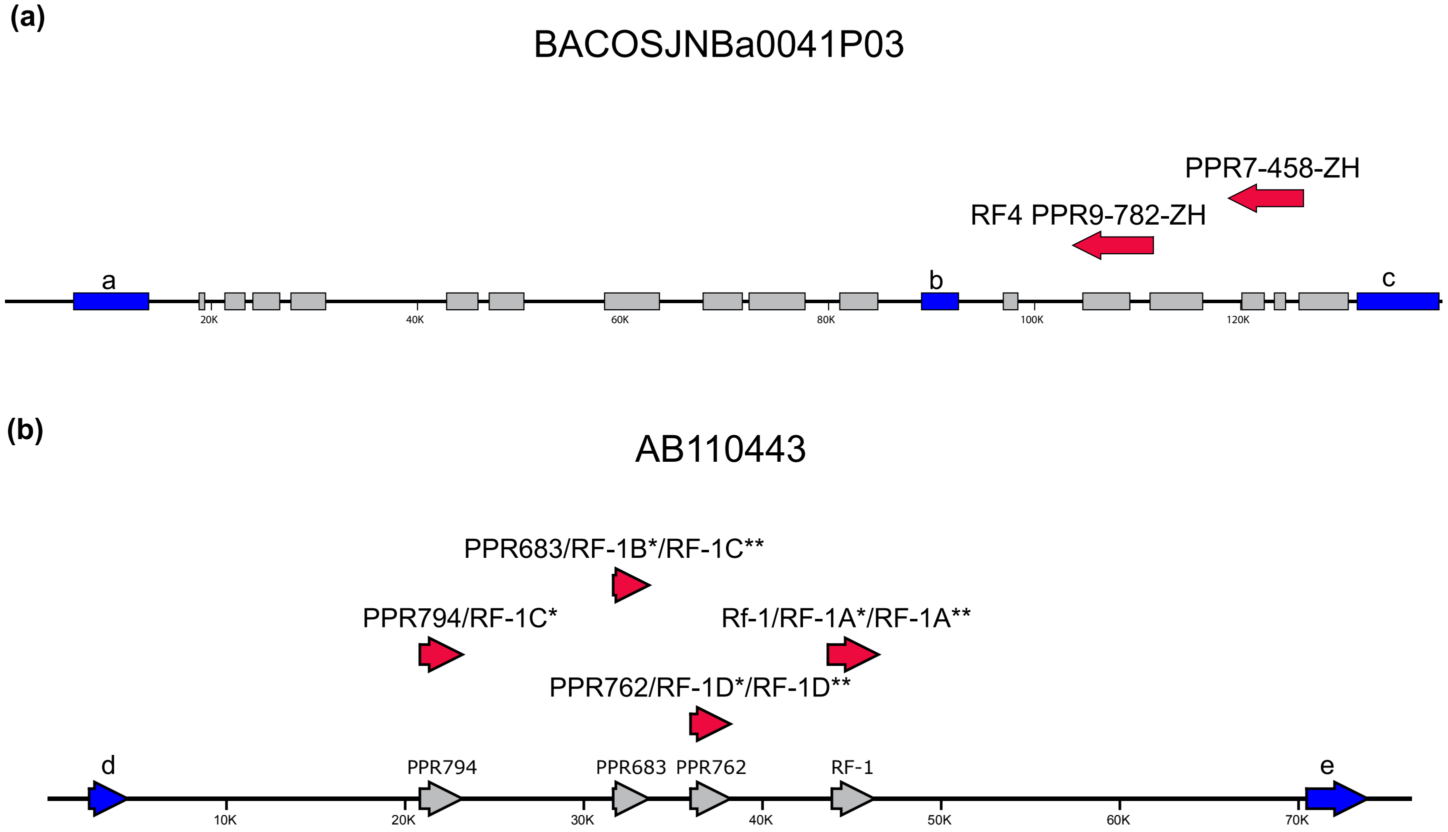

Supplementary Figure S2. Arrangement of *RFL* genes in the *RF-region 1* and *2* as published by Tang *et al.,* 2014 (BACOSJNBa0041P03) and Komori *et al.,* 2004 (AB110443). The DNA sequences were obtained from National Center for Biotechnology Information (NCBI, http://www.ncbi.nlm.nih.gov/) and gene annotations were visualized in KONG Cloning Suite (www.kongcloning.com). * protein assignment as published in Kato *et al.,* 2007; ** this manuscript.

Kato, H. et al. Structural diversity and evolution of the Rf-1 locus in the genus Oryza. Heredity (Edinb) 99, 516-524, doi:10.1038/sj.hdy.6801026 (2007).

Komori, T. et al. Map-based cloning of a fertility restorer gene, Rf-1, in rice (Oryza sativa L.). Plant J 37, 315-325, doi:10.1111/j.1365-313X.2004.01961.x (2004)

Tang, H. W. et al. The Rice Restorer Rf4 for Wild-Abortive Cytoplasmic Male Sterility encodes a mitochondrial-localized PPR Protein that functions in reduction of WA352 transcripts. Mol Plant 7, 1497-1500, doi:10.1093/mp/ssu047 (2014).

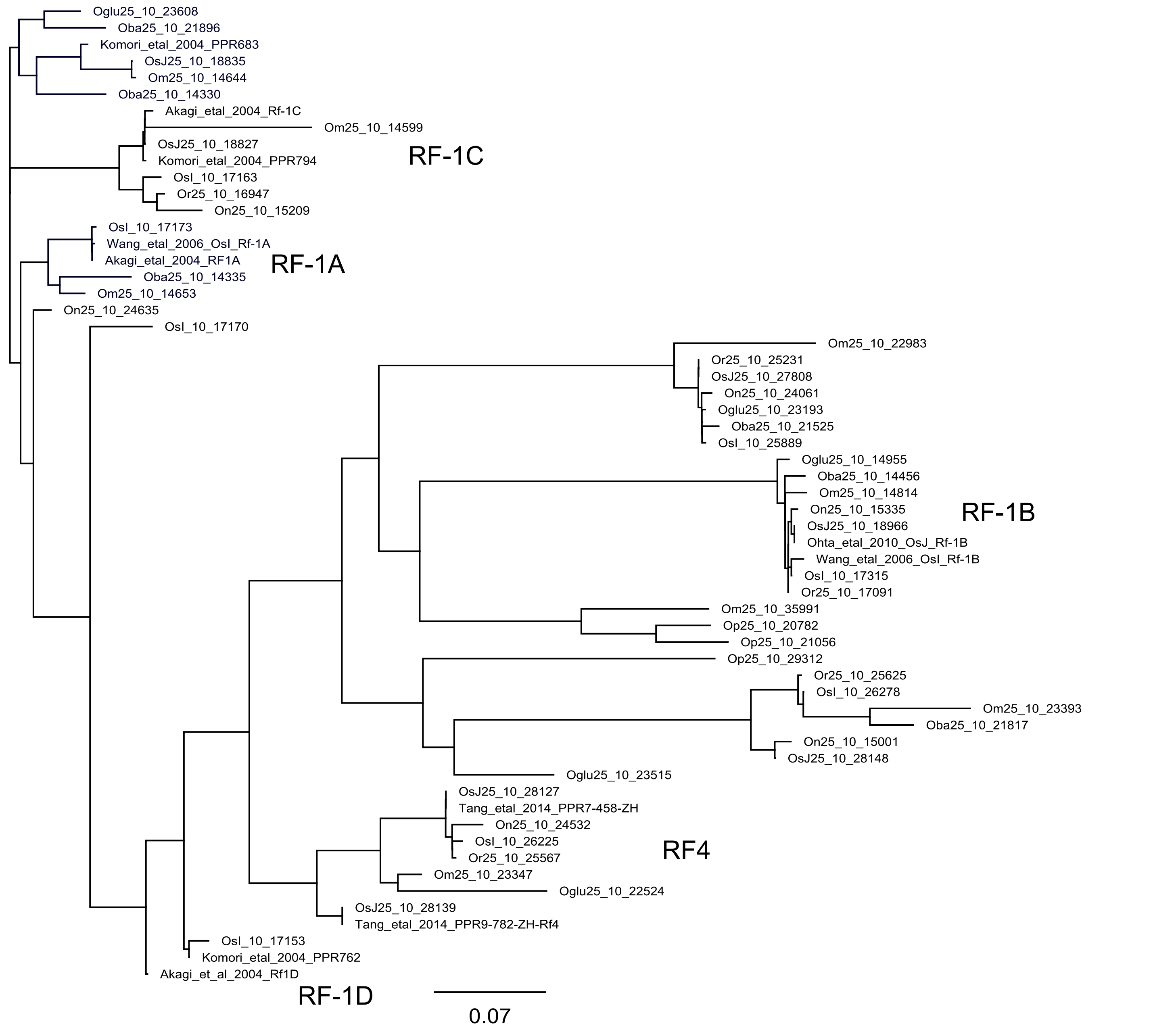

Supplementary Figure S3. Phylogenetic relationships between RFL proteins encoded by genes located within the *RFL* cluster located on chromosome 10 in nine *Oryza* species. The scale bar represents the number of substitutions per site.

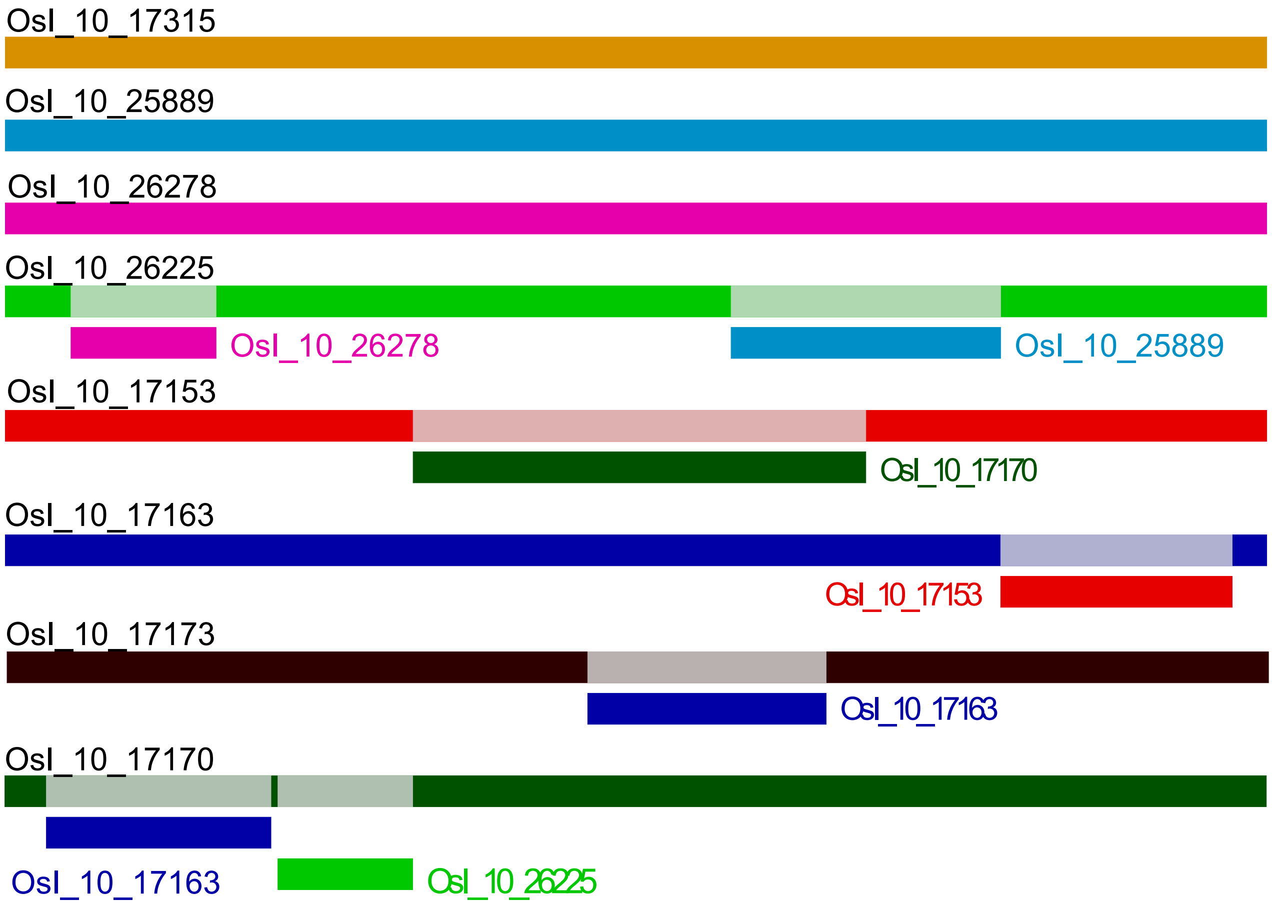

Supplementary Figure S4. Schematic sequence display showing putative recombination events between*RFL* sequences located on *O.sativa* indica chromosome 10. The DNA sequences were aligned with MUSCLE 3.8.31 (Edgar et al., 2004). The resulting seguence alignment was used for prediction of recombination events with the Recombination Detection Program (RDP4) (Martin et al., 2015).

Martin DP, Murrell B, Golden M, Khoosal A, & Muhire B RDP4: Detection and analysis of recombination patterns in virus genomes. Virus Evol, 1: vev003 doi: 10.1093/ve/vev003 (2015).

Edgar, R. C. MUSCLE: multiple sequence alignment with high accuracy and high throughput. Nucleic Acids Res 32, 1792-1797, doi:10.1093/nar/gkh340 (2004).

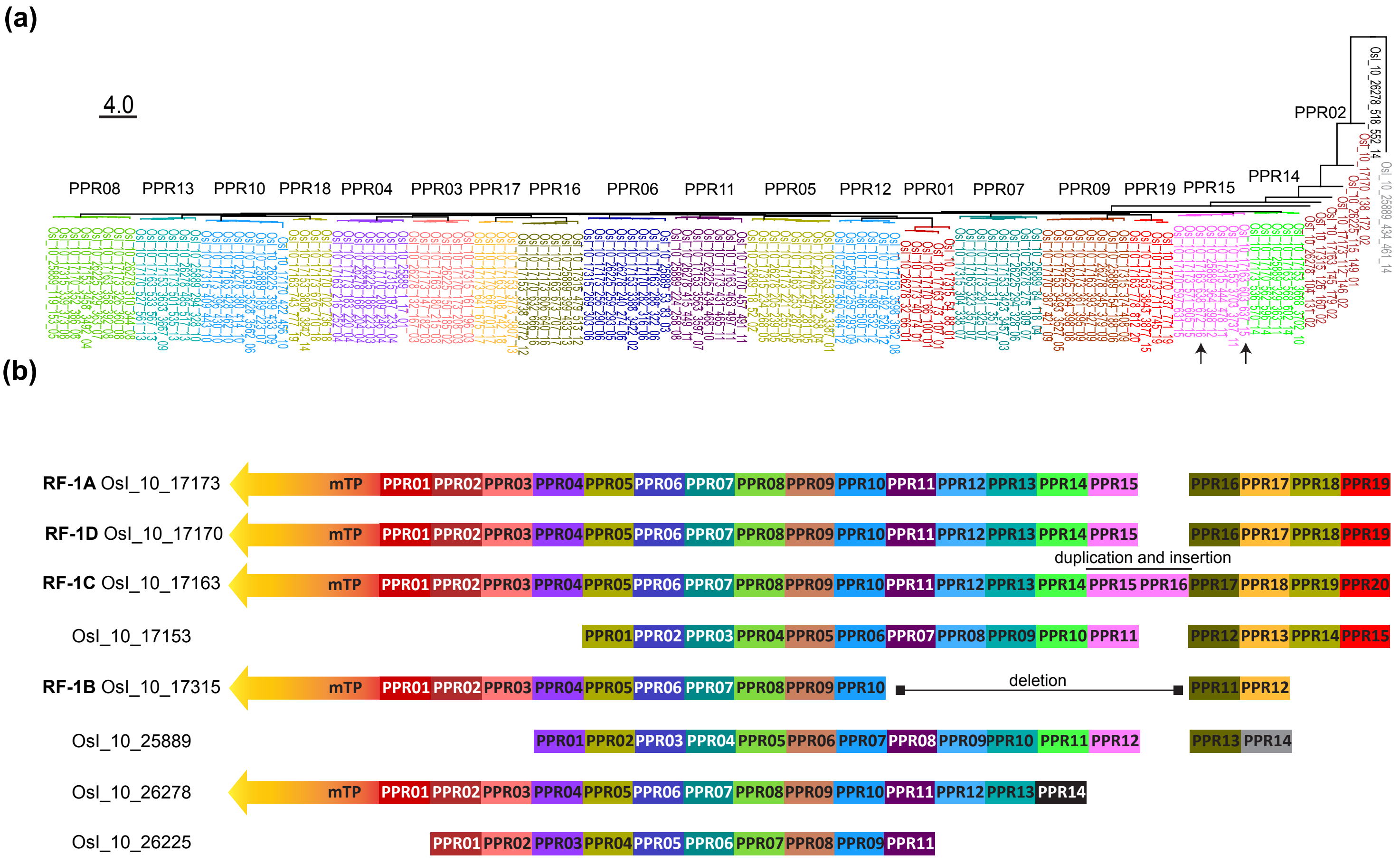

Supplementary Figure S5. Analysis of the PPR motifs in the RFL proteins encoded within the cluster located on chromosome 10 in *Oryza sativa indica*. (**a**) Tree illustrating the phylogenetic relationships between PPR motifs extracted from protein sequences and numbered starting from the amino-terminus is displayed. In total, 123 PPR motifs from 8 RFLproteins were aligned. (**b**) Comparison of the schematic representations illustrating the distribution of the PPR motifs in the sequences of RFL proteins. The PPR motifs are coloured according to the tree shown in (**a**). Duplication and insertaion, as well as deletion of PPR motifs have been indicated.

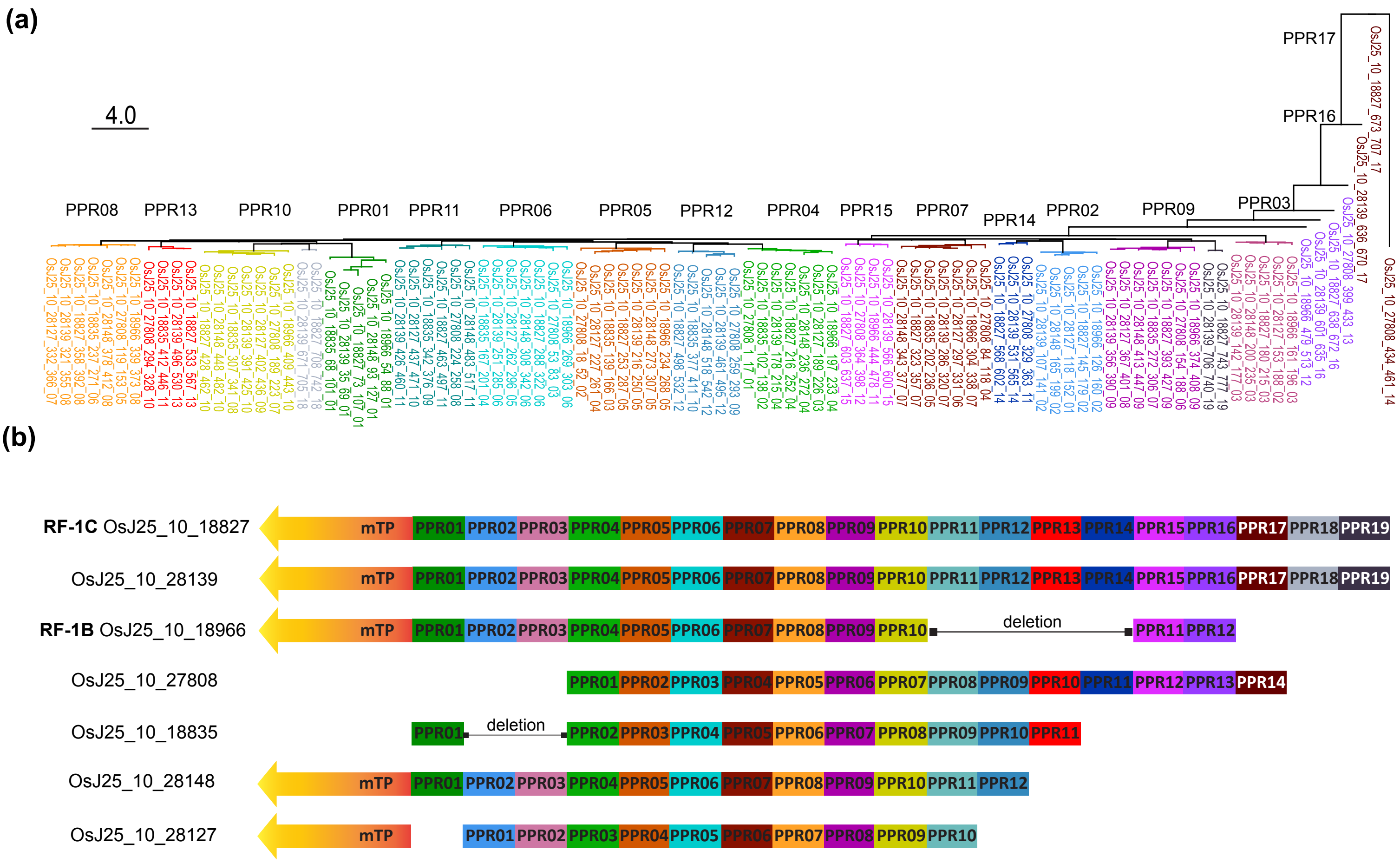

Supplementary Figure S6. Analysis of the PPR motifs in the RFL proteins encoded within the cluster located on chromosome 10 in *Oryza sativa japonica.* (**a**) Tree illustrating the phylogenetic relationships between PPR motifs extracted from protein sequences and numbered starting from the amino-terminus is displayed. In total, 97 PPR motifs from 7 RFL proteins were aligned. (**b**) Comparison of the schematic representations illustrating the distribution of the PPR motifs in the sequences of RFL proteins. The PPR motifs are coloured according to the tree shown in (**a**). Deletions of PPR motifs have been indicated.

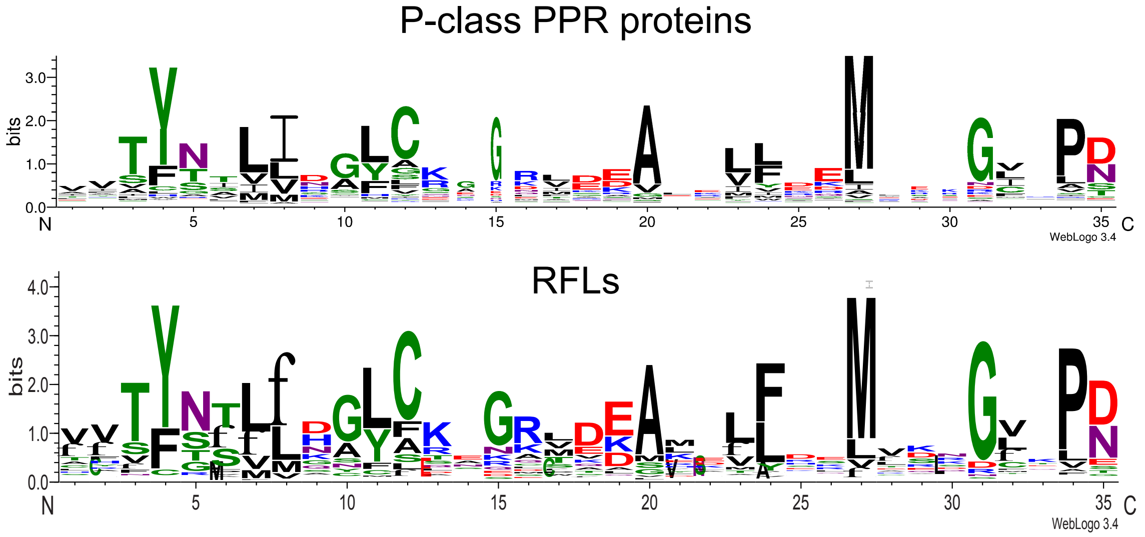

Supplementary Figure S7. PPR motifs sequences were aligned with MAFT and the logos were generated with WebLogo 3.4 (Crooks et al., 2004) (http://weblogo.threeplusone.com/create.cgi). The logos illustrate the amino acid conservation at each position along the PPR motif. Polar amino acids are shown in green, neutral in purple, basic in blue, acidic in red and hydrophobic in black.

Crooks, G. E., Hon, G., Chandonia, J. M. & Brenner, S. E. WebLogo: a sequence logo generator. Genome Res 14, 1188-1190, doi:10.1101/gr.849004 (2004).

Supplementary Table S1. To date published *Oryza* RF and RFL sequences.

Species	Cultivar	NCBI reference r	Gene/protein identifier	Protein name	Gene name	Restorer allele	References
**RF-1**							
Oryza sativa indica group	Milyang 23	AB106867				*Rf-1*	
Oryza sativa indica group	BTR	AB110016				*Rf-1*	Kazama & Toriyama 2003
Oryza sativa indica group	Milyang 23	AB195686		PPR8-3		*Rf-1*	
Oryza sativa japonica group	Taichung 65(T)	AB470408			*rf1a*	*rf-1*	
Oryza sativa japonica group	Taichung 65(T)	AB470409			*rf1b*	*rf-1*	Ohta et al., 2010
Oryza sativa japonica group	Taichung 65(R)	AB470406			*rf1a*	*rf-1*	
Oryza sativa japonica group	Taichung 65(R)	AB470407			*rf1b*	*rf-1*	
Oryza sativa indica group	Minghui63	DQ311053			*Rf1a*	*Rf-1*	
Oryza sativa indica group	Minghui63	DQ311054			*Rf1b*	*Rf-1*	Wang et al., 2006
Oryza sativa indica group	FuyuA	DQ311052			*rf1a*	*rf-1*	
Oryza sativa indica group	MTC-10R	AB179840	BAD20283		*Rf-1A*	*Rf-1*	
Oryza sativa indica group	MTC-10R	AB179840	pseudogene		*Rf-1B*	*Rf-1*	Akagi et al., 2004
Oryza sativa indica group	MTC-10R	AB179840	BAD20284		*Rf-1D*	*Rf-1*	
Oryza sativa indica group	MTC-10R	AB112811	BAD13711		*Rf-1C*	*Rf-1*	
Oryza sativa indica group	IR24	AB110443	BAD08214	RF-1 (PPR791)		*Rf-1*	
Oryza sativa indica group	IR24	AB110443	BAD08211	PPR794		*Rf-1*	
Oryza sativa indica group	IR24	AB110443	BAD08212	PPR683		*Rf-1*	
Oryza sativa indica group	IR24	AB110443	BAD08213	PPR762		*Rf-1*	Komori et al., 2004
Oryza sativa japonica	Asominori	AB110444	BAD08215			*rf-1*	
Oryza sativa japonica	Asominori	AB110444	BAD08216			*rf-1*	
Oryza sativa japonica	Asominori	AB110444	BAD08217			*rf-1*	
**RF4 (86% amino acid similarity to RF-1A (Tang et al., 2014))**							
Oryza sativa indica group	IR24	AB900791		PPR454		*Rf4-I*	
Oryza sativa indica group	IR24	AB900792		PPR782a		*Rf4-I*	Kazama & Toriyama 2014
Oryza sativa indica group	IR24	AB900793		PPR782b		*Rf4-I*	
Oryza sativa indica group	IR24	AB900794		PPR458		*Rf4-I*	
Oryza sativa japonica group	Nipponbare	Os10g0495400	Os10g0495400	PPR7-458-N		*rf4-j*	
Oryza sativa japonica group	Nipponbare	Os10g0495200	Os10g0495200	PPR9-782-N		*rf4-j*	
Oryza sativa japonica group	Nipponbare	Os10g0495100	Os10g0495100	PPR10 -569-N		*rf4-j*	
Oryza sativa japonica group	Zhonghua11	KJ680250		PPR9-782-ZH		*rf4-j*	
Oryza sativa indica group	Minghui63	KJ680249		PPR9-782-M		*Rf4-M*	
Oryza sativa indica group	IR24	KJ680248		PPR9-782-I		*Rf4-I*	
Oryza sativa indica group	Zhenshan97A	KJ680247		PPR9-409-Z		*rf4-i*	
Oryza sativa indica group	93-11	KJ680253		PPR10-469-93		*rf4-i*	Tang et al., 2014
Oryza sativa indica group	Zhenshan97A	KJ680252		PPR10-454-Z		*rf4-i*	
Oryza sativa indica group	Minghui63	KJ680251		PPR10-454-M		*Rf4-M*	
Oryza sativa indica group	Minghui63	KJ680246		PPR8-780-M		*Rf4-M*	
Oryza sativa japonica group	Zhonghua11	KJ680245		PPR7-458-ZH		*rf4-j*	
Oryza sativa indica group	Zhenshan97A	KJ680244		PPR7-458-Z		*rf4-i*	
Oryza sativa indica group	IR24	KJ680243	Os10g0495400	PPR7-458-I		*Rf4-I*	
Oryza sativa indica group	Minghui 63	KJ680242		PPR7-454-M		*Rf4-M*	
**RF5 (identical to RF-1A (Hu et al., 2012))**							
Oryza sativa indica x O. rufipogon	Hong-Lian CMS YueTai A (YTA)	AB179840				*Rf5*	Hu et al., 2012
**RF6**							
Oryza sativa indica x O. rufipogon	Hong-Lian CMS YueTai A (YTA)	AK066315 and AK068841				*Rf6*	Huang et al., 2015

References:

1. Kazama, T. & Toriyama, K. A pentatricopeptide repeat-containing gene that promotes the processing of aberrant atp6 RNA of cytoplasmic male-sterile rice. Febs Lett 544, 99-102, doi:Doi 10.1016/S0014-5793(03)00480-0 (2003). **2.** Ohta, H., Ogino, A., Kasai, M., Sano, Y. & Kanazawa, A. Fertility restoration by Ifr1 in rice with BT-type cytoplasmic male sterility is associated with a reduced level, but not processing, of atp6-orf79 co-transcribed RNA. Plant Cell Rep 29, 359-369, doi:10.1007/s00299-010-0827-7 (2010). **3.** Wang, Z. H. et al. Cytoplasmic male sterility of rice with boro II cytoplasm is caused by a cytotoxic peptide and is restored by two related PPR motif genes via distinct modes of mRNA silencing. Plant Cell 18, 676-687, doi:DOI 10.1105/tpc.105.038240 (2006). **4.** Akagi, H. et al. Positional cloning of the rice Rf-1 gene, a restorer of BT-type cytoplasmic male sterility that encodes a mitochondria-targeting PPR protein. Theoretical and Applied Genetics 108, 1449-1457, doi:DOI 10.1007/s00122-004-1591-2 (2004). **5.** Komori, T. et al. Map-based cloning of a fertility restorer gene, Rf-1, in rice (Oryza sativa L.). Plant Journal 37, 315-325, doi:10.1111/j.1365-313X.2004.01961.x (2004). **6.** Kazama, T. & Toriyama, K. A fertility restorer gene, Rf4, widely used for hybrid rice breeding encodes a pentatricopeptide repeat protein. Rice 7, doi:ARTN 2810.1186/s12284-014-0028-z (2014). **7.** Tang, H. W. et al. The Rice Restorer Rf4 for Wild-Abortive Cytoplasmic Male Sterility Encodes a Mitochondrial-Localized PPR Protein that Functions in Reduction of WA352 Transcripts. Mol Plant 7, 1497-1500, doi:10.1093/mp/ssu047 (2014). **8.** Hu, J. et al. The rice pentatricopeptide repeat protein RF5 restores fertility in Hong-Lian cytoplasmic male-sterile lines via a complex with the glycine-rich protein GRP162. Plant Cell 24, 109-122, doi:10.1105/tpc.111.093211 (2012). **9.** Huang, W. et al. Pentatricopeptide-repeat family protein RF6 functions with hexokinase 6 to rescue rice cytoplasmic male sterility. Proc Natl Acad Sci U S A 112, 14984-14989, doi:10.1073/pnas.1511748112 (2015).

Supplementary Table S2. Summary of data sets used in the study.

Nb	Species	Cultivar	Version genome/ database release	Website	year of release/ update	File name
1	*Oryza sativa japonica*		v1.0	http://plants.ensembl.org/Oryza_sativa/Info/Index	2015	ensemble.Oryza_sativa.japonica.IRGSP-1.0.25.dna.genome.fa.gz
2	*Oryza indica*		v1	http://plants.ensembl.org/Oryza_indica/Info/Index	2015	ensemble.Oryza_indica.ASM465v1.25.dna.genome.fa.gz
3	*Oryza sativa Nipponbare*		IRGSP-1.0	http://rapdb.dna.affrc.go.jp/index.html	2013	rabdb.O_nipponbare.IRGSP-1.0_genome.fasta.gz
4	rice cultivar 'Kasalath'	Kasalath		http://rapdb.dna.affrc.go.jp/index.html	2013	rapdb.O_kasalath_genome.tar
5	*Oryza barthii*		v1.4	http://plants.ensembl.org/Oryza_barthii/Info/Index	2015	ensemble.Oryza_barthii.ABRL00000000.25.dna.genome.fa.gz
6	*Oryza brachyantha*		v1.4	http://plants.ensembl.org/Oryza_brachyantha/Info/Index	2015	ensemble.Oryza_brachyantha.Oryza_brachyantha.v1.4b.25.dna.genome.fa.gz
7	*Oryza glaberrima*		v1.1	http://plants.ensembl.org/Oryza_glaberrima/Info/Index	2015	ensemble.Oryza_glaberrima.AGI1.1.25.dna.genome.fa.gz
8	*Oryza glumaepatula*		release-25	http://plants.ensembl.org/Oryza_glumaepatula/Info/Index	2015	ensemble.Oryza_glumaepatula.ALNU02000000.25.dna.genome.fa.gz
9	*Oryza meridionalis*		release-25	http://plants.ensembl.org/Oryza_meridionalis/Info/Index	2015	ensemble.Oryza_meridionalis.ALNW00000000.25.dna.genome.fa.gz
10	*Oryza nivara*		release-25	http://plants.ensembl.org/Oryza_nivara/Info/Index	2015	ensemble.Oryza_nivara.AWHD00000000.25.dna.genome.fa.gz
11	*Oryza punctata*		release-25	http://plants.ensembl.org/Oryza_punctata/Info/Index	2015	ensemble.Oryza_punctata.AVCL00000000.25.dna.genome.fa.gz
12	*Oryza longistaminata*		v1.30	http://plants.ensembl.org/Oryza_longistaminata/Info/Index	2016	ensemble.Oryza_longistaminata.GCA_000789195.1.30.dna.genome.fa.gz
13	*Oryza rufipogon*		release-25	http://plants.ensembl.org/Oryza_rufipogon/Info/Index	2015	ensemble.Oryza_rufipogon.PRJEB4137.25.dna.genome.fa.gz
14	*Brachypodium distachyon*		v2.1	http://phytozome.jgi.doe.gov/pz/portal.html#!bulk?org=Org_Bdistach	2010	phytozyme.Bdistachyon_283_assembly_v2.0.fa.gz

Supplementary Table S3. Identification of *RFL* sequences in genomic sequence data from 13 *Oryza* genomes and the *Brachypodium distachyon* genome.

Nb	Tribe	Genus	Species	Genomes coded names	Genome data				Identification of RFL proteins						
									Identification ORFs containing PPR motifs			Nb of identified RFL proteins			
					Genome size (Mb)	Ploidy	Genome type	References	Nb of ORFs (x1000)	Nb of ORFs containing PPR repeats	Nb of ORFs containing (>10) P class PPR repeats	OrthoFinder	OrthoMCL	CD-Hit	Phylogeny
1			*Oryza sativa japonica*	OsJ	389	2n=2x=24	AA	1	752	617	129	13	14	13	14
2			*Oryza sativa indica*	OsI	389	2n=2x=24	AA	2	844	719	128	18	18	18	18
3			*Oryza sativa Nipponbare*	OsN	321	2n=2x=24	AA	3	749	617	129	13	14	13	14
4			*Oryza sativa indica, kasalath*	OsK	331	2n=2x=24	AA	3	756	628	116	11	11	10	11
5			*Oryza barthii*	Oba25	411	2n=2x=24	AA	I-OMAP	569	624	125	13	13	12	13
6			*Oryza brachyantha*	Obr25	261	2n=2x=24	FF	4	423	589	111	4	4	0	4
7	*Oryzeae*	*Oryza*	*Oryza glaberrima*	Ogla25	354	2n=2x=24	AA	5	668	570	105	2	2	0	2
8			*Oryza glumaepatula*	Oglu25	464	2n=2x=24	AA	I-OMAP	618	610	124	12	12	11	12
9			*Oryza meridionalis*	Om25	435	2n=2x=24	AA	I-OMAP	722	589	124	14	15	15	15
10			*Oryza nivara*	On25	448	2n=2x=24	AA	I-OMAP	623	619	128	13	13	10	13
11			*Oryza longistaminata*	Olo	352	2n=2x=24	AA	6	591	680	144	12	12	12	12
12			*Oryza punctata*	Op25	423	2n=2x=24	BB	I-OMAP	748	645	123	7	7	7	7
13			*Oryza rufipogon*	Or25	445	2n=2x=24	AA	I-OMAP	679	640	126	12	12	10	12
14	*Brachypodieae*	*Brachypodium*	*Brachypodium distachyon*	Bd21	271	2n=2x=10	n.a	7	663	582	124	10	11	10	10
								**Total:**	**9404**	**8729**	**1736**	**154**	**158**	**141**	**157**
											**Average nb of RFLs:**	**11**	**11**	**10**	**11**

n.a. not annotated
I-OMAP - International Oryza Map Alignment

1. Ouyang, S. et al. The TIGR Rice Genome Annotation Resource: Improvements and new features. Nucleic Acids Res 35, D883-D887, doi:10.1093/nar/gkl976 (2007). **2.** Kawahara, Y. et al. Improvement of the Oryza sativa Nipponbare reference genome using next generation sequence and optical map data. Rice 6, doi:Artn 410.1186/1939-8433-6-4 (2013). **3.** Sakai, H. et al. Rice Annotation Project Database (RAP-DB): An Integrative and Interactive Database for Rice Genomics. Plant Cell Physiol 54, E6-+, doi:10.1093/pcp/pcs183 (2013). Project. **4.** Chen, J. F. et al. Whole-genome sequencing of Oryza brachyantha reveals mechanisms underlying Oryza genome evolution. Nature Communications 4, doi:ARTN 159510.1038/ncomms2596 (2013). **5.** Wang, M. H. et al. The genome sequence of African rice (Oryza glaberrima) and evidence for independent domestication. Nat Genet 46, 982-+, doi:10.1038/ng.3044 (2014). **6.** Zhang Y, Zhang S, Liu H, Fu B, Li L, Xie M, Song Y, Li X, Cai J, Wan W, Kui L, Huang H, Lyu J, Dong Y, Wang W, Huang L, Zhang J, Yang Q, Shan Q, Li Q, Huang W, Tao D, Wang M, Chen M, Yu Y, Wing RA, Wang W, Hu F (2015) Genome and Comparative Transcriptomics of African Wild Rice Oryza longistaminata Provide Insights into Molecular Mechanism of Rhizomatousness and Self-Incompatibility. Mol Plant. 2015 Nov 2;8(11):1683-6. doi: 10.1016/j.molp.2015.08.006. **7.** International Brachypodium, I. Genome sequencing and analysis of the model grass Brachypodium distachyon. Nature 463, 763-768, doi:10.1038/nature08747 (2010).

Supplementary Table S4. Assignment of P-class PPR protein sequences into orthologous groups by OrthoMCL and OrthoFinder, and clusters by CD-Hit. RF-orthogroups/cluster are highlighted in red.

OrthoFinder			OrthoMCL			CD-Hit (OsJ)	
Orthologous group	# of proteins	Average number of PPR proteins per plant species	Orthologous group	# of proteins	Average number of PPR proteins per plant species	Cluster	# of proteins
OG000000:	134	9.57	1	194	13.86	1	13
OG000001:	59	4.21	2	67	4.79	2	4
OG000002:	27	1.93	3	60	4.29	3	2
OG000003:	19	1.36	4	58	4.14	4	2
OG000004:	16	1.14	5	27	1.93	5	2
OG000005:	16	1.14	6	24	1.71	6	2
OG000006:	15	1.07	7	19	1.36	7	1
OG000007:	15	1.07	8	16	1.14	8	1
OG000008:	15	1.07	9	16	1.14	9	1
OG000009:	15	1.07	10	15	1.07	10	1
OG000010:	15	1.07	11	15	1.07	11	1
OG000011:	15	1.07	12	15	1.07	12	1
OG000012:	15	1.07	13	15	1.07	13	1
OG000013:	15	1.07	14	15	1.07	14	1
OG000014:	15	1.07	15	15	1.07	15	1
OG000015:	15	1.07	16	15	1.07	16	1
OG000016:	15	1.07	17	14	1.00	17	1
OG000017:	14	1.00	18	14	1.00	18	1
OG000018:	14	1.00	19	14	1.00	19	1
OG000019:	14	1.00	20	14	1.00	20	1
OG000020:	14	1.00	21	14	1.00	21	1
OG000021:	14	1.00	22	14	1.00	22	1
OG000022:	14	1.00	23	14	1.00	23	1
OG000023:	14	1.00	24	14	1.00	24	1
OG000024:	14	1.00	25	14	1.00	25	1
OG000025:	14	1.00	26	14	1.00	26	1
OG000026:	14	1.00	27	14	1.00	27	1
OG000027:	14	1.00	28	14	1.00	28	1
OG000028:	14	1.00	29	14	1.00	29	1
OG000029:	14	1.00	30	14	1.00	30	1
OG000030:	14	1.00	31	14	1.00	31	1
OG000031:	14	1.00	32	14	1.00	32	1
OG000032:	14	1.00	33	14	1.00	33	1
OG000033:	14	1.00	34	14	1.00	34	1
OG000034:	14	1.00	35	14	1.00	35	1
OG000035:	14	1.00	36	14	1.00	36	1
OG000036:	14	1.00	37	14	1.00	37	1
OG000037:	14	1.00	38	14	1.00	38	1
OG000038:	14	1.00	39	14	1.00	39	1
OG000039:	14	1.00	40	14	1.00	40	1
OG000040:	14	1.00	41	14	1.00	41	1
OG000041:	14	1.00	42	14	1.00	42	1
OG000042:	14	1.00	43	14	1.00	43	1
OG000043:	14	1.00	44	14	1.00	44	1
OG000044:	14	1.00	45	14	1.00	45	1
OG000045:	14	1.00	46	14	1.00	46	1
OG000046:	14	1.00	47	14	1.00	47	1
OG000047:	14	1.00	48	14	1.00	48	1
OG000048:	14	1.00	49	14	1.00	49	1
OG000049:	14	1.00	50	14	1.00	50	1
OG000050:	14	1.00	51	14	1.00	51	1
OG000051:	14	1.00	52	14	1.00	52	1
OG000052:	14	1.00	53	14	1.00	53	1
OG000053:	14	1.00	54	14	1.00	54	1
OG000054:	14	1.00	55	14	1.00	55	1
OG000055:	14	1.00	56	14	1.00	56	1
OG000056:	14	1.00	57	14	1.00	57	1
OG000057:	14	1.00	58	14	1.00	58	1
OG000058:	14	1.00	59	14	1.00	59	1
OG000059:	14	1.00	60	14	1.00	60	1
OG000060:	14	1.00	61	14	1.00	61	1
OG000061:	14	1.00	62	14	1.00	62	1
OG000062:	14	1.00	63	14	1.00	63	1
OG000063:	14	1.00	64	14	1.00	64	1
OG000064:	14	1.00	65	14	1.00	65	1
OG000065:	14	1.00	66	14	1.00	66	1
OG000066:	14	1.00	67	13	0.93	67	1
OG000067:	14	1.00	68	13	0.93	68	1
OG000068:	14	1.00	69	13	0.93	69	1
OG000069:	14	1.00	70	13	0.93	70	1
OG000070:	14	1.00	71	13	0.93	71	1
OG000071:	14	1.00	72	13	0.93	72	1
OG000072:	13	0.93	73	13	0.93	73	1
OG000073:	13	0.93	74	13	0.93	74	1
OG000074:	13	0.93	75	13	0.93	75	1
OG000075:	13	0.93	76	13	0.93	76	1
OG000076:	13	0.93	77	13	0.93	77	1
OG000077:	13	0.93	78	13	0.93	78	1
OG000078:	13	0.93	79	13	0.93	79	1
OG000079:	13	0.93	80	13	0.93	80	1
OG000080:	13	0.93	81	13	0.93	81	1
OG000081:	13	0.93	82	13	0.93	82	1
OG000082:	13	0.93	83	13	0.93	83	1
OG000083:	13	0.93	84	13	0.93	84	1
OG000084:	13	0.93	85	13	0.93	85	1
OG000085:	13	0.93	86	13	0.93	86	1
OG000086:	13	0.93	87	13	0.93	87	1
OG000087:	13	0.93	88	13	0.93	88	1
OG000088:	13	0.93	89	13	0.93	89	1
OG000089:	13	0.93	90	13	0.93	90	1
OG000090:	13	0.93	91	13	0.93	91	1
OG000091:	13	0.93	92	13	0.93	92	1
OG000092:	13	0.93	93	12	0.86	93	1
OG000093:	13	0.93	94	12	0.86	94	1
OG000094:	13	0.93	95	12	0.86	95	1
OG000095:	13	0.93	96	12	0.86	96	1
OG000096:	13	0.93	97	12	0.86	97	1
OG000097:	13	0.93	98	12	0.86	98	1
OG000098:	13	0.93	99	12	0.86	99	1
OG000099:	13	0.93	100	11	0.79	100	1
OG000100:	13	0.93	101	11	0.79	101	1
OG000101:	13	0.93	102	10	0.71	102	1
OG000102:	12	0.86				103	1
OG000103:	12	0.86				104	1
OG000104:	12	0.86				105	1
OG000105:	12	0.86				106	1
OG000106:	12	0.86				107	1
OG000107:	12	0.86				108	1
OG000108:	12	0.86				109	1
OG000109:	12	0.86				110	1
OG000110:	11	0.79					

Supplementary Table S5. List of RFL sequences assigned to RFL-clade by analysis with OrthoFinder, OrthoMCL-DB, CD-Hit and Phylogeny. Sequences of the reference RFLs (Fuji et al., 2011) are highlighted in red.

OrthoFinder	OrthoMCL-DB	CD-Hit	Phylogeny
Bd21_Bd1_12685	Bd21_Bd1_12685	Bd21_Bd1_12685	Bd21_Bd1_12685
Bd21_Bd2_49161	Bd21_Bd2_49161	Bd21_Bd2_49161	Bd21_Bd2_49161
Bd21_Bd2_96705	Bd21_Bd2_96705	Bd21_Bd2_96705	Bd21_Bd2_96705
Bd21_Bd2_96716	Bd21_Bd2_96716	Bd21_Bd2_96716	Bd21_Bd2_96716
Bd21_Bd2_96721	Bd21_Bd2_96721	Bd21_Bd2_96721	Bd21_Bd2_96721
Bd21_Bd2_96765	Bd21_Bd2_96765	Bd21_Bd2_96765	Bd21_Bd2_96765
Bd21_Bd2_97034	Bd21_Bd2_97034	Bd21_Bd2_97034	Bd21_Bd2_97034
Bd21_Bd2_97043	Bd21_Bd2_97043	Bd21_Bd2_97043	Bd21_Bd2_97043
Bd21_Bd3_19755	Bd21_Bd2_9844	Bd21_Bd3_19755	Bd21_Bd3_19755
Bd21_Bd4_112460	Bd21_Bd3_19755	Bd21_Bd4_112460	Bd21_Bd4_112460
Bradi1G14190	Bd21_Bd4_112460	Oba25_10_14330	Bradi1G14190
Bradi2G38110	Bradi1G14190	Oba25_10_14335	Bradi2G38110
Bradi2G38120	Bradi2G38110	Oba25_10_14456	Bradi2G38120
Bradi2G38400	Bradi2G38120	Oba25_10_21525	Bradi2G38400
Bradi2G38440	Bradi2G38400	Oba25_10_21817	Bradi2G38440
Bradi2G38450	Bradi2G38440	Oba25_10_21896	Bradi2G38450
Bradi2G38460	Bradi2G38450	Oba25_4_9472	Bradi2G38460
Bradi2g38760	Bradi2G38460	Oba25_4_9514	Bradi2g38760
Oba25_10_14330	Bradi2g38760	Oba25_8_40119	Bradi4g05910
Oba25_10_14335	Bradi4g05910	Oba25_8_40135	Oba25_10_14330
Oba25_10_14456	Oba25_10_14330	Oba25_8_418	Oba25_10_14335
Oba25_10_21525	Oba25_10_14335	Oba25_8_5649	Oba25_10_14456
Oba25_10_21817	Oba25_10_14456	Oglu25_10_22524	Oba25_10_21525
Oba25_10_21896	Oba25_10_21525	Oglu25_10_23193	Oba25_10_21817
Oba25_4_9455	Oba25_10_21817	Oglu25_10_23515	Oba25_10_21896
Oba25_4_9472	Oba25_10_21896	Oglu25_10_23608	Oba25_4_9455
Oba25_4_9514	Oba25_4_9455	Oglu25_4_10677	Oba25_4_9472
Oba25_8_40119	Oba25_4_9472	Oglu25_4_10697	Oba25_4_9514
Oba25_8_40135	Oba25_4_9514	Oglu25_4_10744	Oba25_8_40119
Oba25_8_418	Oba25_8_40119	Oglu25_8_43214	Oba25_8_40135
Oba25_8_5649	Oba25_8_40135	Oglu25_8_43229	Oba25_8_418
Obr25_4_31256	Oba25_8_418	Oglu25_8_449	Oba25_8_5649
Obr25_4_5174	Oba25_8_5649	Oglu25_8_6078	Obr25_4_31256
Obr25_7_19230	Obr25_4_31256	Olo_K538760_1_316	Obr25_4_5174
Obr25_8_28251	Obr25_4_5174	Olo_K538760_1_430	Obr25_7_19230
Ogla25_8_118	Obr25_7_19230	Olo_K538777_1_447	Obr25_8_28251
Ogla25_8_6391	Obr25_8_28251	Olo_K538777_1_468	Ogla25_8_118
Oglu25_10_14955	Ogla25_8_118	Olo_K538809_1_154	Ogla25_8_6391
Oglu25_10_22524	Ogla25_8_6391	Olo_K538809_1_175	Oglu25_10_14955
Oglu25_10_23193	Oglu25_10_14955	Olo_K538908_1_31	Oglu25_10_22524
Oglu25_10_23515	Oglu25_10_22524	Olo_K538911_1_217	Oglu25_10_23193
Oglu25_10_23608	Oglu25_10_23193	Olo_K539297_1_199	Oglu25_10_23515
Oglu25_4_10677	Oglu25_10_23515	Olo_K539297_1_211	Oglu25_10_23608
Oglu25_4_10697	Oglu25_10_23608	Olo_K539297_1_212	Oglu25_4_10677
Oglu25_4_10744	Oglu25_4_10677	Olo_K541232_1_10	Oglu25_4_10697
Oglu25_8_43214	Oglu25_4_10697	Om25_10_14599	Oglu25_4_10744
Oglu25_8_43229	Oglu25_4_10744	Om25_10_14644	Oglu25_8_43214
Oglu25_8_449	Oglu25_8_43214	Om25_10_14653	Oglu25_8_43229
Oglu25_8_6078	Oglu25_8_43229	Om25_10_14814	Oglu25_8_449
Olo_KN538760_1_316	Oglu25_8_449	Om25_10_22983	Oglu25_8_6078
Olo_KN538760_1_430	Oglu25_8_6078	Om25_10_23347	Olo_KN538760_1_316
Olo_KN538777_1_447	Olo_KN538760_1_316	Om25_10_23393	Olo_KN538760_1_430
Olo_KN538777_1_468	Olo_KN538760_1_430	Om25_10_35991	Olo_KN538777_1_447
Olo_KN538809_1_154	Olo_KN538777_1_447	Om25_4_12487	Olo_KN538777_1_468
Olo_KN538809_1_175	Olo_KN538777_1_468	Om25_4_12510	Olo_KN538809_1_154
Olo_KN538908_1_31	Olo_KN538809_1_154	Om25_4_12518	Olo_KN538809_1_175
Olo_KN538911_1_217	Olo_KN538809_1_175	Om25_4_12609	Olo_KN538908_1_31
Olo_KN539297_1_199	Olo_KN538908_1_31	Om25_8_41181	Olo_KN538911_1_217
Olo_KN539297_1_211	Olo_KN538911_1_217	Om25_8_44833	Olo_KN539297_1_199
Olo_KN539297_1_212	Olo_KN539297_1_199	Om25_8_44852	Olo_KN539297_1_211
Olo_KN541232_1_10	Olo_KN539297_1_211	On25_10_15335	Olo_KN539297_1_212
Om25_10_14599	Olo_KN539297_1_212	On25_10_24061	Olo_KN541232_1_10
Om25_10_14653	Olo_KN541232_1_10	On25_10_24532	Om25_10_14599
Om25_10_14814	Om25_10_14599	On25_10_24635	Om25_10_14644
Om25_10_22983	Om25_10_14644	On25_4_8505	Om25_10_14653
Om25_10_23347	Om25_10_14653	On25_4_8553	Om25_10_14814
Om25_10_23393	Om25_10_14814	On25_8_431	Om25_10_22983
Om25_10_35991	Om25_10_22983	On25_8_47555	Om25_10_23347
Om25_4_12487	Om25_10_23347	On25_8_47566	Om25_10_23393
Om25_4_12510	Om25_10_23393	On25_8_7032	Om25_10_35991
Om25_4_12518	Om25_10_35991	Op25_10_20782	Om25_4_12487
Om25_4_12609	Om25_4_12487	Op25_10_21056	Om25_4_12510
Om25_8_41181	Om25_4_12510	Op25_10_29312	Om25_4_12518
Om25_8_44833	Om25_4_12518	Op25_4_13412	Om25_4_12609
Om25_8_44852	Om25_4_12609	Op25_4_13477	Om25_8_41181
On25_10_15001	Om25_8_41181	Op25_8_54025	Om25_8_44833
On25_10_15209	Om25_8_44833	Op25_8_585	Om25_8_44852
On25_10_15335	Om25_8_44852	Or25_10_16947	On25_10_15001
On25_10_24061	On25_10_15001	Or25_10_17091	On25_10_15209
On25_10_24532	On25_10_15209	Or25_10_25231	On25_10_15335
On25_10_24635	On25_10_15335	Or25_10_25567	On25_10_24061
On25_4_8484	On25_10_24061	Or25_10_25625	On25_10_24532
On25_4_8505	On25_10_24532	Or25_4_13645	On25_10_24635
On25_4_8553	On25_10_24635	Or25_8_424	On25_4_8484
On25_8_431	On25_4_8484	Or25_8_51062	On25_4_8505
On25_8_47555	On25_4_8505	Or25_8_51070	On25_4_8553
On25_8_47566	On25_4_8553	Or25_8_7591	On25_8_431
On25_8_7032	On25_8_431	Osl_1_13674	On25_8_47555
Op25_10_20782	On25_8_47555	Osl_10_17153	On25_8_47566
Op25_10_21056	On25_8_47566	Osl_10_17163	On25_8_7032
Op25_10_29312	On25_8_7032	Osl_10_17170	Op25_10_20782
Op25_4_13412	Op25_10_20782	Osl_10_17173	Op25_10_21056
Op25_4_13477	Op25_10_21056	Osl_10_17315	Op25_10_29312
Op25_8_54025	Op25_10_29312	Osl_10_25889	Op25_4_13412
Op25_8_585	Op25_4_13412	Osl_10_26225	Op25_4_13477
Or25_10_16947	Op25_4_13477	Osl_10_26278	Op25_8_54025

Or25_10_17091	Op25_8_54025	OsI_3_7625	Op25_8_585
Or25_10_25231	Op25_8_585	OsI_4_13106	Or25_10_16947
Or25_10_25567	Or25_10_16947	OsI_4_13129	Or25_10_17091
Or25_10_25625	Or25_10_17091	OsI_4_54892	Or25_10_25231
Or25_4_13645	Or25_10_25231	OsI_8_518	Or25_10_25567
Or25_4_13666	Or25_10_25567	OsI_8_56093	Or25_10_25625
Or25_4_13729	Or25_10_25625	OsI_8_56135	Or25_4_13645
Or25_8_424	Or25_4_13645	OsI_8_8415	Or25_4_13666
Or25_8_51062	Or25_4_13666	OsI_8_8433	Or25_4_13729
Or25_8_51070	Or25_4_13729	OsJ25_10_18827	Or25_8_424
Or25_8_7591	Or25_8_424	OsJ25_10_18835	Or25_8_51062
OS04G28234	Or25_8_51062	OsJ25_10_18966	Or25_8_51070
OS04G28300	Or25_8_51070	OsJ25_10_27808	Or25_8_7591
OS08G01640	Or25_8_7591	OsJ25_10_28127	OS04G28234
OS08G01650	OS04G28234	OsJ25_10_28139	OS04G28300
OS08G01870	OS04G28300	OsJ25_10_28148	OS08G01640
OS08g15000	OS08G01640	OsJ25_4_17214	OS08G01650
OS10g35230	OS08G01650	OsJ25_4_17272	OS08G01870
OS10G35240	OS08G01870	OsJ25_8_431	OS08g15000
Os10g35260	OS08g15000	OsJ25_8_55568	OS10g35230
Os10g35440	OS10g35230	OsJ25_8_55582	OS10G35240
Os10g35640	OS10G35240	OsJ25_8_8485	Os10g35260
OsI_1_13674	Os10g35260	OsK_chr04_10933	Os10g35440
OsI_10_17153	Os10g35440	OsK_chr04_10951	Os10g35640
OsI_10_17163	Os10g35640	OsK_chr08_431	OsI_1_13674
OsI_10_17170	OsI_1_13674	OsK_chr08_46716	OsI_10_17153
OsI_10_17173	OsI_10_17153	OsK_chr08_46727	OsI_10_17163
OsI_10_17315	OsI_10_17163	OsK_chr08_6608	OsI_10_17170
OsI_10_25889	OsI_10_17170	OsK_chr10_15274	OsI_10_17173
OsI_10_26225	OsI_10_17173	OsK_chr10_15421	OsI_10_17315
OsI_10_26278	OsI_10_17315	OsK_chr10_23474	OsI_10_25889
OsI_3_7625	OsI_10_25889	OsK_UM_16352	OsI_10_26225
OsI_4_13106	OsI_10_26225	OsN_chr04_17214	OsI_10_26278
OsI_4_13129	OsI_10_26278	OsN_chr04_17272	OsI_3_7625
OsI_4_54892	OsI_3_7625	OsN_chr08_431	OsI_4_13106
OsI_8_518	OsI_4_13106	OsN_chr08_55568	OsI_4_13129
OsI_8_56093	OsI_4_13129	OsN_chr08_55582	OsI_4_54892
OsI_8_56135	OsI_4_54892	OsN_chr08_8485	OsI_8_518
OsI_8_8415	OsI_8_518	OsN_chr10_18827	OsI_8_56093
OsI_8_8433	OsI_8_56093	OsN_chr10_18835	OsI_8_56135
OsJ25_10_18827	OsI_8_56135	OsN_chr10_18966	OsI_8_8415
OsJ25_10_18966	OsI_8_8415	OsN_chr10_27808	OsI_8_8433
OsJ25_10_27808	OsI_8_8433	OsN_chr10_28127	OsJ25_10_18827
OsJ25_10_28127	OsJ25_10_18827	OsN_chr10_28139	OsJ25_10_18835
OsJ25_10_28139	OsJ25_10_18835	OsN_chr10_28148	OsJ25_10_18966
OsJ25_10_28148	OsJ25_10_18966		OsJ25_10_27808
OsJ25_4_17195	OsJ25_10_27808		OsJ25_10_28127
OsJ25_4_17214	OsJ25_10_28127		OsJ25_10_28139
OsJ25_4_17272	OsJ25_10_28139		OsJ25_10_28148
OsJ25_8_431	OsJ25_10_28148		OsJ25_4_17195
OsJ25_8_55568	OsJ25_4_17195		OsJ25_4_17214
OsJ25_8_55582	OsJ25_4_17214		OsJ25_4_17272
OsJ25_8_8485	OsJ25_4_17272		OsJ25_8_431
OsK_chr04_10933	OsJ25_8_431		OsJ25_8_55568
OsK_chr04_10951	OsJ25_8_55568		OsJ25_8_55582
OsK_chr04_10997	OsJ25_8_55582		OsJ25_8_8485
OsK_chr08_431	OsJ25_8_8485		OsK_chr04_10933
OsK_chr08_46716	OsK_chr04_10933		OsK_chr04_10951
OsK_chr08_46727	OsK_chr04_10951		OsK_chr04_10997
OsK_chr08_6608	OsK_chr04_10997		OsK_chr08_431
OsK_chr10_15274	OsK_chr08_431		OsK_chr08_46716
OsK_chr10_15421	OsK_chr08_46716		OsK_chr08_46727
OsK_chr10_23474	OsK_chr08_46727		OsK_chr08_6608
OsK_UM_16352	OsK_chr08_6608		OsK_chr10_15274
OsN_chr04_17195	OsK_chr10_15274		OsK_chr10_15421
OsN_chr04_17214	OsK_chr10_15421		OsK_chr10_23474
OsN_chr04_17272	OsK_chr10_23474		OsK_UM_16352
OsN_chr08_431	OsK_UM_16352		OsN_chr04_17195
OsN_chr08_55568	OsN_chr04_17195		OsN_chr04_17214
OsN_chr08_55582	OsN_chr04_17214		OsN_chr04_17272
OsN_chr08_8485	OsN_chr04_17272		OsN_chr08_431
OsN_chr10_18827	OsN_chr08_431		OsN_chr08_55568
OsN_chr10_18966	OsN_chr08_55568		OsN_chr08_55582
OsN_chr10_27808	OsN_chr08_55582		OsN_chr08_8485
OsN_chr10_28127	OsN_chr08_8485		OsN_chr10_18827
OsN_chr10_28139	OsN_chr10_18827		OsN_chr10_18835
OsN_chr10_28148	OsN_chr10_18835		OsN_chr10_18966
OsRf1a	OsN_chr10_18966		OsN_chr10_27808
OsRF1b	OsN_chr10_27808		OsN_chr10_28127
OsRFL10l	OsN_chr10_28127		OsN_chr10_28139
OsRFL11l	OsN_chr10_28139		OsN_chr10_28148
OsRFL12l	OsN_chr10_28148		OsRf1a
OsRFL13l	OsRf1a		OsRf1b
OsRFL14i	OsRf1b		OsRFL10l
OsRFL15l	OsRFL10l		OsRFL11l
OsRFL16l	OsRFL11l		OsRFL12l
OsRFL1l	OsRFL12l		OsRFL13l
OsRFL2l	OsRFL13l		OsRFL14i
OsRFL3i	OsRFL14i		OsRFL15l
OsRFL4l	OsRFL15l		OsRFL16l
OsRFL5i	OsRFL16l		OsRFL1l
OsRFL7i	OsRFL1l		OsRFL2l
OsRFL8l	OsRFL2l		OsRFL3i
	OsRFL3i		OsRFL4l
	OsRFL4l		OsRFL5i
	OsRFL5i		OsRFL7i
	OsRFL7i		OsRFL8l
	OsRFL8l		

Supplementary Table S6. Sequence overlap between the four methods used for the identification of RFL PPR proteins.

OrthoFinder	OrthoFinder	OrthoMCL	CD-hit	Phylogeny
Bd21_Bd1_12685	yes	yes	yes	yes
Bd21_Bd2_49161	yes	yes	yes	yes
Bd21_Bd2_96705	yes	yes	yes	yes
Bd21_Bd2_96716	yes	yes	yes	yes
Bd21_Bd2_96721	yes	yes	yes	yes
Bd21_Bd2_96765	yes	yes	yes	yes
Bd21_Bd2_97034	yes	yes	yes	yes
Bd21_Bd2_97043	yes	yes	yes	yes
Bd21_Bd3_19755	yes	yes	yes	yes
Bd21_Bd4_112460	yes	yes	yes	yes
Oba25_10_14330	yes	yes	yes	yes
Oba25_10_14335	yes	yes	yes	yes
Oba25_10_14456	yes	yes	yes	yes
Oba25_10_21525	yes	yes	yes	yes
Oba25_10_21817	yes	yes	yes	yes
Oba25_10_21896	yes	yes	yes	yes
Oba25_4_9472	yes	yes	yes	yes
Oba25_4_9514	yes	yes	yes	yes
Oba25_8_40119	yes	yes	yes	yes
Oba25_8_40135	yes	yes	yes	yes
Oba25_8_418	yes	yes	yes	yes
Oba25_8_5649	yes	yes	yes	yes
Oglu25_10_22524	yes	yes	yes	yes
Oglu25_10_23193	yes	yes	yes	yes
Oglu25_10_23515	yes	yes	yes	yes
Oglu25_10_23608	yes	yes	yes	yes
Oglu25_4_10677	yes	yes	yes	yes
Oglu25_4_10697	yes	yes	yes	yes
Oglu25_4_10744	yes	yes	yes	yes
Oglu25_8_43214	yes	yes	yes	yes
Oglu25_8_43229	yes	yes	yes	yes
Oglu25_8_449	yes	yes	yes	yes
Oglu25_8_6078	yes	yes	yes	yes
Om25_10_14599	yes	yes	yes	yes
Om25_10_14653	yes	yes	yes	yes
Om25_10_14814	yes	yes	yes	yes
Om25_10_22983	yes	yes	yes	yes
Om25_10_23347	yes	yes	yes	yes
Om25_10_23393	yes	yes	yes	yes
Om25_10_35991	yes	yes	yes	yes
Om25_4_12487	yes	yes	yes	yes
Om25_4_12510	yes	yes	yes	yes
Om25_4_12518	yes	yes	yes	yes
Om25_4_12609	yes	yes	yes	yes
Om25_8_41181	yes	yes	yes	yes
Om25_8_44833	yes	yes	yes	yes
Om25_8_44852	yes	yes	yes	yes
On25_10_15335	yes	yes	yes	yes
On25_10_24061	yes	yes	yes	yes
On25_10_24532	yes	yes	yes	yes
On25_10_24635	yes	yes	yes	yes
On25_4_8505	yes	yes	yes	yes
On25_4_8553	yes	yes	yes	yes
On25_8_431	yes	yes	yes	yes
On25_8_47555	yes	yes	yes	yes
On25_8_47566	yes	yes	yes	yes
On25_8_7032	yes	yes	yes	yes
Op25_10_20782	yes	yes	yes	yes
Op25_10_21056	yes	yes	yes	yes
Op25_10_29312	yes	yes	yes	yes
Op25_4_13412	yes	yes	yes	yes
Op25_4_13477	yes	yes	yes	yes
Op25_8_54025	yes	yes	yes	yes
Op25_8_585	yes	yes	yes	yes
Or25_10_16947	yes	yes	yes	yes
Or25_10_17091	yes	yes	yes	yes
Or25_10_25231	yes	yes	yes	yes
Or25_10_25567	yes	yes	yes	yes
Or25_10_25625	yes	yes	yes	yes
Or25_4_13645	yes	yes	yes	yes
Or25_8_424	yes	yes	yes	yes
Or25_8_51062	yes	yes	yes	yes
Or25_8_51070	yes	yes	yes	yes
Or25_8_7591	yes	yes	yes	yes
OsI_1_13674	yes	yes	yes	yes
OsI_10_17153	yes	yes	yes	yes
OsI_10_17163	yes	yes	yes	yes
OsI_10_17170	yes	yes	yes	yes
OsI_10_17173	yes	yes	yes	yes
OsI_10_17315	yes	yes	yes	yes
OsI_10_25889	yes	yes	yes	yes
OsI_10_26225	yes	yes	yes	yes
OsI_10_26278	yes	yes	yes	yes
OsI_3_7625	yes	yes	yes	yes
OsI_4_13106	yes	yes	yes	yes
OsI_4_13129	yes	yes	yes	yes
OsI_4_54892	yes	yes	yes	yes
OsI_8_518	yes	yes	yes	yes
OsI_8_56093	yes	yes	yes	yes
OsI_8_56135	yes	yes	yes	yes
OsI_8_8415	yes	yes	yes	yes
OsI_8_8433	yes	yes	yes	yes
OsJ25_10_18827	yes	yes	yes	yes
OsJ25_10_18966	yes	yes	yes	yes
OsJ25_10_27808	yes	yes	yes	yes
OsJ25_10_28127	yes	yes	yes	yes
OsJ25_10_28139	yes	yes	yes	yes
OsJ25_10_28148	yes	yes	yes	yes
OsJ25_4_17214	yes	yes	yes	yes
OsJ25_4_17272	yes	yes	yes	yes
OsJ25_8_431	yes	yes	yes	yes
OsJ25_8_55568	yes	yes	yes	yes
OsJ25_8_55582	yes	yes	yes	yes
OsJ25_8_8485	yes	yes	yes	yes
OsK_chr04_10933	yes	yes	yes	yes
OsK_chr04_10951	yes	yes	yes	yes
OsK_chr08_431	yes	yes	yes	yes
OsK_chr08_46716	yes	yes	yes	yes
OsK_chr08_46727	yes	yes	yes	yes
OsK_chr08_6608	yes	yes	yes	yes
OsK_chr10_15274	yes	yes	yes	yes
OsK_chr10_15421	yes	yes	yes	yes
OsK_chr10_23474	yes	yes	yes	yes
OsK_UM_16352	yes	yes	yes	yes
OsN_chr04_17214	yes	yes	yes	yes
OsN_chr04_17272	yes	yes	yes	yes
OsN_chr08_431	yes	yes	yes	yes
OsN_chr08_55568	yes	yes	yes	yes
OsN_chr08_55582	yes	yes	yes	yes
OsN_chr08_8485	yes	yes	yes	yes
OsN_chr10_18827	yes	yes	yes	yes
OsN_chr10_18966	yes	yes	yes	yes
OsN_chr10_27808	yes	yes	yes	yes
OsN_chr10_28127	yes	yes	yes	yes
OsN_chr10_28139	yes	yes	yes	yes
OsN_chr10_28148	yes	yes	yes	yes
Olo_KN538760_1_316	yes	yes	yes	yes
Olo_KN538760_1_430	yes	yes	yes	yes
Olo_KN538777_1_447	yes	yes	yes	yes
Olo_KN538777_1_468	yes	yes	yes	yes
Olo_KN538809_1_154	yes	yes	yes	yes
Olo_KN538809_1_175	yes	yes	yes	yes
Olo_KN538908_1_31	yes	yes	yes	yes
Olo_KN538911_1_217	yes	yes	yes	yes
Olo_KN539297_1_199	yes	yes	yes	yes
Olo_KN539297_1_211	yes	yes	yes	yes
Olo_KN539297_1_212	yes	yes	yes	yes
Olo_KN541232_1_10	yes	yes	yes	yes
Oba25_4_9455	yes	yes	#N/A	yes
Obr25_4_31256	yes	yes	#N/A	yes
Obr25_4_5174	yes	yes	#N/A	yes
Obr25_7_19230	yes	yes	#N/A	yes
Obr25_8_28251	yes	yes	#N/A	yes
Ogla25_8_118	yes	yes	#N/A	yes
Ogla25_8_6391	yes	yes	#N/A	yes
Oglu25_10_14955	yes	yes	#N/A	yes
On25_10_15001	yes	yes	#N/A	yes
On25_10_15209	yes	yes	#N/A	yes
On25_4_8484	yes	yes	#N/A	yes
Or25_4_13666	yes	yes	#N/A	yes
Or25_4_13729	yes	yes	#N/A	yes
OsJ25_4_17195	yes	yes	#N/A	yes
OsK_chr04_10997	yes	yes	#N/A	yes
OsN_chr04_17195	yes	yes	#N/A	yes

OrthoMCL	OrthoMCL	OrthoFinder	CD-hit	Phylogeny
Bd21_Bd1_12685	yes	yes	yes	yes
Bd21_Bd2_49161	yes	yes	yes	yes
Bd21_Bd2_96705	yes	yes	yes	yes
Bd21_Bd2_96716	yes	yes	yes	yes
Bd21_Bd2_96721	yes	yes	yes	yes
Bd21_Bd2_96765	yes	yes	yes	yes
Bd21_Bd2_97034	yes	yes	yes	yes
Bd21_Bd2_97043	yes	yes	yes	yes
Bd21_Bd3_19755	yes	yes	yes	yes
Bd21_Bd4_112460	yes	yes	yes	yes
Oba25_10_14330	yes	yes	yes	yes
Oba25_10_14335	yes	yes	yes	yes
Oba25_10_14456	yes	yes	yes	yes
Oba25_10_21525	yes	yes	yes	yes
Oba25_10_21817	yes	yes	yes	yes
Oba25_10_21896	yes	yes	yes	yes
Oba25_4_9472	yes	yes	yes	yes
Oba25_4_9514	yes	yes	yes	yes
Oba25_8_40119	yes	yes	yes	yes
Oba25_8_40135	yes	yes	yes	yes
Oba25_8_418	yes	yes	yes	yes
Oba25_8_5649	yes	yes	yes	yes
Oglu25_10_22524	yes	yes	yes	yes
Oglu25_10_23193	yes	yes	yes	yes
Oglu25_10_23515	yes	yes	yes	yes
Oglu25_10_23608	yes	yes	yes	yes
Oglu25_4_10677	yes	yes	yes	yes
Oglu25_4_10697	yes	yes	yes	yes
Oglu25_4_10744	yes	yes	yes	yes
Oglu25_8_43214	yes	yes	yes	yes
Oglu25_8_43229	yes	yes	yes	yes
Oglu25_8_449	yes	yes	yes	yes
Oglu25_8_6078	yes	yes	yes	yes
Olo_KN538760_1_316	yes	yes	yes	yes
Olo_KN538760_1_430	yes	yes	yes	yes
Olo_KN538777_1_447	yes	yes	yes	yes
Olo_KN538777_1_468	yes	yes	yes	yes
Olo_KN538809_1_154	yes	yes	yes	yes
Olo_KN538809_1_175	yes	yes	yes	yes
Olo_KN538908_1_31	yes	yes	yes	yes
Olo_KN538911_1_217	yes	yes	yes	yes
Olo_KN539297_1_199	yes	yes	yes	yes
Olo_KN539297_1_211	yes	yes	yes	yes
Olo_KN539297_1_212	yes	yes	yes	yes
Olo_KN541232_1_10	yes	yes	yes	yes
Om25_10_14599	yes	yes	yes	yes
Om25_10_14653	yes	yes	yes	yes
Om25_10_14814	yes	yes	yes	yes
Om25_10_22983	yes	yes	yes	yes
Om25_10_23347	yes	yes	yes	yes
Om25_10_23393	yes	yes	yes	yes
Om25_10_35991	yes	yes	yes	yes
Om25_4_12487	yes	yes	yes	yes
Om25_4_12510	yes	yes	yes	yes
Om25_4_12518	yes	yes	yes	yes
Om25_4_12609	yes	yes	yes	yes
Om25_8_41181	yes	yes	yes	yes
Om25_8_44833	yes	yes	yes	yes
Om25_8_44852	yes	yes	yes	yes
On25_10_15335	yes	yes	yes	yes
On25_10_24061	yes	yes	yes	yes
On25_10_24532	yes	yes	yes	yes
On25_10_24635	yes	yes	yes	yes
On25_4_8505	yes	yes	yes	yes
On25_4_8553	yes	yes	yes	yes
On25_8_431	yes	yes	yes	yes
On25_8_47555	yes	yes	yes	yes
On25_8_47566	yes	yes	yes	yes
On25_8_7032	yes	yes	yes	yes
Op25_10_20782	yes	yes	yes	yes
Op25_10_21056	yes	yes	yes	yes
Op25_10_29312	yes	yes	yes	yes
Op25_4_13412	yes	yes	yes	yes
Op25_4_13477	yes	yes	yes	yes
Op25_8_54025	yes	yes	yes	yes
Op25_8_585	yes	yes	yes	yes
Or25_10_16947	yes	yes	yes	yes
Or25_10_17091	yes	yes	yes	yes
Or25_10_25231	yes	yes	yes	yes
Or25_10_25567	yes	yes	yes	yes
Or25_10_25625	yes	yes	yes	yes
Or25_4_13645	yes	yes	yes	yes
Or25_8_424	yes	yes	yes	yes
Or25_8_51062	yes	yes	yes	yes
Or25_8_51070	yes	yes	yes	yes
Or25_8_7591	yes	yes	yes	yes
OsI_1_13674	yes	yes	yes	yes
OsI_10_17153	yes	yes	yes	yes
OsI_10_17163	yes	yes	yes	yes
OsI_10_17170	yes	yes	yes	yes
OsI_10_17173	yes	yes	yes	yes
OsI_10_17315	yes	yes	yes	yes
OsI_10_25889	yes	yes	yes	yes
OsI_10_26225	yes	yes	yes	yes
OsI_10_26278	yes	yes	yes	yes
OsI_3_7625	yes	yes	yes	yes
OsI_4_13106	yes	yes	yes	yes
OsI_4_13129	yes	yes	yes	yes
OsI_4_54892	yes	yes	yes	yes
OsI_8_518	yes	yes	yes	yes
OsI_8_56093	yes	yes	yes	yes
OsI_8_56135	yes	yes	yes	yes
OsI_8_8415	yes	yes	yes	yes
OsI_8_8433	yes	yes	yes	yes
OsJ25_10_18827	yes	yes	yes	yes
OsJ25_10_18966	yes	yes	yes	yes
OsJ25_10_27808	yes	yes	yes	yes
OsJ25_10_28127	yes	yes	yes	yes
OsJ25_10_28139	yes	yes	yes	yes
OsJ25_10_28148	yes	yes	yes	yes
OsJ25_4_17214	yes	yes	yes	yes
OsJ25_4_17272	yes	yes	yes	yes
OsJ25_8_431	yes	yes	yes	yes
OsJ25_8_55568	yes	yes	yes	yes
OsJ25_8_55582	yes	yes	yes	yes
OsJ25_8_8485	yes	yes	yes	yes
OsK_chr04_10933	yes	yes	yes	yes
OsK_chr04_10951	yes	yes	yes	yes
OsK_chr08_431	yes	yes	yes	yes
OsK_chr08_46716	yes	yes	yes	yes
OsK_chr08_46727	yes	yes	yes	yes
OsK_chr08_6608	yes	yes	yes	yes
OsK_chr10_15274	yes	yes	yes	yes
OsK_chr10_15421	yes	yes	yes	yes
OsK_chr10_23474	yes	yes	yes	yes
OsK_UM_16352	yes	yes	yes	yes
OsN_chr04_17214	yes	yes	yes	yes
OsN_chr04_17272	yes	yes	yes	yes
OsN_chr08_431	yes	yes	yes	yes
OsN_chr08_55568	yes	yes	yes	yes
OsN_chr08_55582	yes	yes	yes	yes
OsN_chr08_8485	yes	yes	yes	yes
OsN_chr10_18827	yes	yes	yes	yes
OsN_chr10_18966	yes	yes	yes	yes
OsN_chr10_27808	yes	yes	yes	yes
OsN_chr10_28127	yes	yes	yes	yes
OsN_chr10_28139	yes	yes	yes	yes
OsN_chr10_28148	yes	yes	yes	yes
Oba25_4_9455	yes	yes	no	yes
Obr25_4_31256	yes	yes	no	yes
Obr25_4_5174	yes	yes	no	yes
Obr25_7_19230	yes	yes	no	yes
Obr25_8_28251	yes	yes	no	yes
Ogla25_8_118	yes	yes	no	yes
Ogla25_8_6391	yes	yes	no	yes
Oglu25_10_14955	yes	yes	no	yes
On25_10_15001	yes	yes	no	yes
On25_10_15209	yes	yes	no	yes
On25_4_8484	yes	yes	no	yes
Or25_4_13666	yes	yes	no	yes
Or25_4_13729	yes	yes	no	yes
OsJ25_4_17195	yes	yes	no	yes
OsK_chr04_10997	yes	yes	no	yes
OsN_chr04_17195	yes	yes	no	yes
Om25_10_14644	yes	no	yes	yes
OsJ25_10_18835	yes	no	yes	yes
OsN_chr10_18835	yes	no	yes	yes
Bd21_Bd2_9844	yes	no	no	no

CD-hit	OrthoMCL	OrthoFinder	Cd-hit	Phylogeny
Bd21_Bd1_12685	yes	yes	yes	yes
Bd21_Bd2_49161	yes	yes	yes	yes
Bd21_Bd2_96705	yes	yes	yes	yes
Bd21_Bd2_96716	yes	yes	yes	yes
Bd21_Bd2_96721	yes	yes	yes	yes
Bd21_Bd2_96765	yes	yes	yes	yes
Bd21_Bd2_97034	yes	yes	yes	yes
Bd21_Bd2_97043	yes	yes	yes	yes
Bd21_Bd3_19755	yes	yes	yes	yes
Bd21_Bd4_112460	yes	yes	yes	yes
Oba25_10_14330	yes	yes	yes	yes
Oba25_10_14335	yes	yes	yes	yes
Oba25_10_14456	yes	yes	yes	yes
Oba25_10_21525	yes	yes	yes	yes
Oba25_10_21817	yes	yes	yes	yes
Oba25_10_21896	yes	yes	yes	yes
Oba25_4_9472	yes	yes	yes	yes
Oba25_4_9514	yes	yes	yes	yes
Oba25_8_40119	yes	yes	yes	yes
Oba25_8_40135	yes	yes	yes	yes
Oba25_8_418	yes	yes	yes	yes
Oba25_8_5649	yes	yes	yes	yes
Oglu25_10_22524	yes	yes	yes	yes
Oglu25_10_23193	yes	yes	yes	yes
Oglu25_10_23515	yes	yes	yes	yes
Oglu25_10_23608	yes	yes	yes	yes
Oglu25_4_10677	yes	yes	yes	yes
Oglu25_4_10697	yes	yes	yes	yes
Oglu25_4_10744	yes	yes	yes	yes
Oglu25_8_43214	yes	yes	yes	yes
Oglu25_8_43229	yes	yes	yes	yes
Oglu25_8_449	yes	yes	yes	yes
Oglu25_8_6078	yes	yes	yes	yes
Olo_KN538760_1_316	yes	yes	yes	yes
Olo_KN538760_1_430	yes	yes	yes	yes
Olo_KN538777_1_447	yes	yes	yes	yes
Olo_KN538777_1_468	yes	yes	yes	yes
Olo_KN538809_1_154	yes	yes	yes	yes
Olo_KN538809_1_175	yes	yes	yes	yes
Olo_KN538908_1_31	yes	yes	yes	yes
Olo_KN538911_1_217	yes	yes	yes	yes
Olo_KN539297_1_199	yes	yes	yes	yes
Olo_KN539297_1_211	yes	yes	yes	yes
Olo_KN539297_1_212	yes	yes	yes	yes
Olo_KN541232_1_10	yes	yes	yes	yes
Om25_10_14599	yes	yes	yes	yes
Om25_10_14653	yes	yes	yes	yes
Om25_10_14814	yes	yes	yes	yes
Om25_10_22983	yes	yes	yes	yes
Om25_10_23347	yes	yes	yes	yes
Om25_10_23393	yes	yes	yes	yes
Om25_10_35991	yes	yes	yes	yes
Om25_4_12487	yes	yes	yes	yes
Om25_4_12510	yes	yes	yes	yes
Om25_4_12518	yes	yes	yes	yes
Om25_4_12609	yes	yes	yes	yes
Om25_8_41181	yes	yes	yes	yes
Om25_8_44833	yes	yes	yes	yes
Om25_8_44852	yes	yes	yes	yes
On25_10_15335	yes	yes	yes	yes
On25_10_24061	yes	yes	yes	yes
On25_10_24532	yes	yes	yes	yes
On25_10_24635	yes	yes	yes	yes
On25_4_8505	yes	yes	yes	yes
On25_4_8553	yes	yes	yes	yes
On25_8_431	yes	yes	yes	yes
On25_8_47555	yes	yes	yes	yes
On25_8_47566	yes	yes	yes	yes
On25_8_7032	yes	yes	yes	yes
Op25_10_20782	yes	yes	yes	yes
Op25_10_21056	yes	yes	yes	yes
Op25_10_29312	yes	yes	yes	yes
Op25_4_13412	yes	yes	yes	yes
Op25_4_13477	yes	yes	yes	yes
Op25_8_54025	yes	yes	yes	yes
Op25_8_585	yes	yes	yes	yes
Or25_10_16947	yes	yes	yes	yes
Or25_10_17091	yes	yes	yes	yes
Or25_10_25231	yes	yes	yes	yes
Or25_10_25567	yes	yes	yes	yes
Or25_10_25625	yes	yes	yes	yes
Or25_4_13645	yes	yes	yes	yes
Or25_8_424	yes	yes	yes	yes
Or25_8_51062	yes	yes	yes	yes
Or25_8_51070	yes	yes	yes	yes
Or25_8_7591	yes	yes	yes	yes
OsI_1_13674	yes	yes	yes	yes
OsI_10_17153	yes	yes	yes	yes
OsI_10_17163	yes	yes	yes	yes
OsI_10_17170	yes	yes	yes	yes
OsI_10_17173	yes	yes	yes	yes
OsI_10_17315	yes	yes	yes	yes
OsI_10_25889	yes	yes	yes	yes
OsI_10_26225	yes	yes	yes	yes
OsI_10_26278	yes	yes	yes	yes
OsI_3_7625	yes	yes	yes	yes
OsI_4_13106	yes	yes	yes	yes
OsI_4_13129	yes	yes	yes	yes
OsI_4_54892	yes	yes	yes	yes
OsI_8_518	yes	yes	yes	yes
OsI_8_56093	yes	yes	yes	yes
OsI_8_56135	yes	yes	yes	yes
OsI_8_8415	yes	yes	yes	yes
OsI_8_8433	yes	yes	yes	yes
OsJ25_10_18827	yes	yes	yes	yes
OsJ25_10_18966	yes	yes	yes	yes
OsJ25_10_27808	yes	yes	yes	yes
OsJ25_10_28127	yes	yes	yes	yes
OsJ25_10_28139	yes	yes	yes	yes
OsJ25_10_28148	yes	yes	yes	yes
OsJ25_4_17214	yes	yes	yes	yes
OsJ25_4_17272	yes	yes	yes	yes
OsJ25_8_431	yes	yes	yes	yes
OsJ25_8_55568	yes	yes	yes	yes
OsJ25_8_55582	yes	yes	yes	yes
OsJ25_8_8485	yes	yes	yes	yes
OsK_chr04_10933	yes	yes	yes	yes
OsK_chr04_10951	yes	yes	yes	yes
OsK_chr08_431	yes	yes	yes	yes
OsK_chr08_46716	yes	yes	yes	yes
OsK_chr08_46727	yes	yes	yes	yes
OsK_chr08_6608	yes	yes	yes	yes
OsK_chr10_15274	yes	yes	yes	yes
OsK_chr10_15421	yes	yes	yes	yes
OsK_chr10_23474	yes	yes	yes	yes
OsK_UM_16352	yes	yes	yes	yes
OsN_chr04_17214	yes	yes	yes	yes
OsN_chr04_17272	yes	yes	yes	yes
OsN_chr08_431	yes	yes	yes	yes
OsN_chr08_55568	yes	yes	yes	yes
OsN_chr08_55582	yes	yes	yes	yes
OsN_chr08_8485	yes	yes	yes	yes
OsN_chr10_18827	yes	yes	yes	yes
OsN_chr10_18966	yes	yes	yes	yes
OsN_chr10_27808	yes	yes	yes	yes
OsN_chr10_28127	yes	yes	yes	yes
OsN_chr10_28139	yes	yes	yes	yes
OsN_chr10_28148	yes	yes	yes	yes
Om25_10_14644	yes	no	yes	yes
OsJ25_10_18835	yes	no	yes	yes
OsN_chr10_18835	yes	no	yes	yes

Phylogeny	OrthoMCL	OrthoFinde	CD-hit	Phylogeny
Bd21_Bd1_12685	yes	yes	yes	yes
Bd21_Bd2_49161	yes	yes	yes	yes
Bd21_Bd2_96705	yes	yes	yes	yes
Bd21_Bd2_96716	yes	yes	yes	yes
Bd21_Bd2_96721	yes	yes	yes	yes
Bd21_Bd2_96765	yes	yes	yes	yes
Bd21_Bd2_97034	yes	yes	yes	yes
Bd21_Bd2_97043	yes	yes	yes	yes
Bd21_Bd3_19755	yes	yes	yes	yes
Bd21_Bd4_112460	yes	yes	yes	yes
Oba25_10_14330	yes	yes	yes	yes
Oba25_10_14335	yes	yes	yes	yes
Oba25_10_14456	yes	yes	yes	yes
Oba25_10_21525	yes	yes	yes	yes
Oba25_10_21817	yes	yes	yes	yes
Oba25_10_21896	yes	yes	yes	yes
Oba25_4_9472	yes	yes	yes	yes
Oba25_4_9514	yes	yes	yes	yes
Oba25_8_40119	yes	yes	yes	yes
Oba25_8_40135	yes	yes	yes	yes
Oba25_8_418	yes	yes	yes	yes
Oba25_8_5649	yes	yes	yes	yes
Oglu25_10_22524	yes	yes	yes	yes
Oglu25_10_23193	yes	yes	yes	yes
Oglu25_10_23515	yes	yes	yes	yes
Oglu25_10_23608	yes	yes	yes	yes
Oglu25_4_10677	yes	yes	yes	yes
Oglu25_4_10697	yes	yes	yes	yes
Oglu25_4_10744	yes	yes	yes	yes
Oglu25_8_43214	yes	yes	yes	yes
Oglu25_8_43229	yes	yes	yes	yes
Oglu25_8_449	yes	yes	yes	yes
Oglu25_8_6078	yes	yes	yes	yes
Olo_KN538760_1_316	yes	yes	yes	yes
Olo_KN538760_1_430	yes	yes	yes	yes
Olo_KN538777_1_447	yes	yes	yes	yes
Olo_KN538777_1_468	yes	yes	yes	yes
Olo_KN538809_1_154	yes	yes	yes	yes
Olo_KN538809_1_175	yes	yes	yes	yes
Olo_KN538908_1_31	yes	yes	yes	yes
Olo_KN538911_1_217	yes	yes	yes	yes
Olo_KN539297_1_199	yes	yes	yes	yes
Olo_KN539297_1_211	yes	yes	yes	yes
Olo_KN539297_1_212	yes	yes	yes	yes
Olo_KN541232_1_10	yes	yes	yes	yes
Om25_10_14599	yes	yes	yes	yes
Om25_10_14653	yes	yes	yes	yes
Om25_10_14814	yes	yes	yes	yes
Om25_10_22983	yes	yes	yes	yes
Om25_10_23347	yes	yes	yes	yes
Om25_10_23393	yes	yes	yes	yes
Om25_10_35991	yes	yes	yes	yes
Om25_4_12487	yes	yes	yes	yes
Om25_4_12510	yes	yes	yes	yes
Om25_4_12518	yes	yes	yes	yes
Om25_4_12609	yes	yes	yes	yes
Om25_8_41181	yes	yes	yes	yes
Om25_8_44833	yes	yes	yes	yes
Om25_8_44852	yes	yes	yes	yes
On25_10_15335	yes	yes	yes	yes
On25_10_24061	yes	yes	yes	yes
On25_10_24532	yes	yes	yes	yes
On25_10_24635	yes	yes	yes	yes
On25_4_8505	yes	yes	yes	yes
On25_4_8553	yes	yes	yes	yes
On25_8_431	yes	yes	yes	yes
On25_8_47555	yes	yes	yes	yes
On25_8_47566	yes	yes	yes	yes
On25_8_7032	yes	yes	yes	yes
Op25_10_20782	yes	yes	yes	yes
Op25_10_21056	yes	yes	yes	yes
Op25_10_29312	yes	yes	yes	yes
Op25_4_13412	yes	yes	yes	yes
Op25_4_13477	yes	yes	yes	yes
Op25_8_54025	yes	yes	yes	yes
Op25_8_585	yes	yes	yes	yes
Or25_10_16947	yes	yes	yes	yes
Or25_10_17091	yes	yes	yes	yes
Or25_10_25231	yes	yes	yes	yes
Or25_10_25567	yes	yes	yes	yes
Or25_10_25625	yes	yes	yes	yes
Or25_4_13645	yes	yes	yes	yes
Or25_8_424	yes	yes	yes	yes
Or25_8_51062	yes	yes	yes	yes
Or25_8_51070	yes	yes	yes	yes
Or25_8_7591	yes	yes	yes	yes
OsI_1_13674	yes	yes	yes	yes
OsI_10_17153	yes	yes	yes	yes
OsI_10_17163	yes	yes	yes	yes
OsI_10_17170	yes	yes	yes	yes
OsI_10_17173	yes	yes	yes	yes
OsI_10_17315	yes	yes	yes	yes
OsI_10_25889	yes	yes	yes	yes
OsI_10_26225	yes	yes	yes	yes
OsI_10_26278	yes	yes	yes	yes
OsI_3_7625	yes	yes	yes	yes
OsI_4_13106	yes	yes	yes	yes
OsI_4_13129	yes	yes	yes	yes
OsI_4_54892	yes	yes	yes	yes
OsI_8_518	yes	yes	yes	yes
OsI_8_56093	yes	yes	yes	yes
OsI_8_56135	yes	yes	yes	yes
OsI_8_8415	yes	yes	yes	yes
OsI_8_8433	yes	yes	yes	yes
OsJ25_10_18827	yes	yes	yes	yes
OsJ25_10_18966	yes	yes	yes	yes
OsJ25_10_27808	yes	yes	yes	yes
OsJ25_10_28127	yes	yes	yes	yes
OsJ25_10_28139	yes	yes	yes	yes
OsJ25_10_28148	yes	yes	yes	yes
OsJ25_4_17214	yes	yes	yes	yes
OsJ25_4_17272	yes	yes	yes	yes
OsJ25_8_431	yes	yes	yes	yes
OsJ25_8_55568	yes	yes	yes	yes
OsJ25_8_55582	yes	yes	yes	yes
OsJ25_8_8485	yes	yes	yes	yes
OsK_chr04_10933	yes	yes	yes	yes
OsK_chr04_10951	yes	yes	yes	yes
OsK_chr08_431	yes	yes	yes	yes
OsK_chr08_46716	yes	yes	yes	yes
OsK_chr08_46727	yes	yes	yes	yes
OsK_chr08_6608	yes	yes	yes	yes
OsK_chr10_15274	yes	yes	yes	yes
OsK_chr10_15421	yes	yes	yes	yes
OsK_chr10_23474	yes	yes	yes	yes
OsK_UM_16352	yes	yes	yes	yes
OsN_chr04_17214	yes	yes	yes	yes
OsN_chr04_17272	yes	yes	yes	yes
OsN_chr08_431	yes	yes	yes	yes
OsN_chr08_55568	yes	yes	yes	yes
OsN_chr08_55582	yes	yes	yes	yes
OsN_chr08_8485	yes	yes	yes	yes
OsN_chr10_18827	yes	yes	yes	yes
OsN_chr10_18966	yes	yes	yes	yes
OsN_chr10_27808	yes	yes	yes	yes
OsN_chr10_28127	yes	yes	yes	yes
OsN_chr10_28139	yes	yes	yes	yes
OsN_chr10_28148	yes	yes	yes	yes
Oba25_4_9455	yes	yes	no	yes
Obr25_4_31256	yes	yes	no	yes
Obr25_4_5174	yes	yes	no	yes
Obr25_7_19230	yes	yes	no	yes
Obr25_8_28251	yes	yes	no	yes
Ogla25_8_118	yes	yes	no	yes
Ogla25_8_6391	yes	yes	no	yes
Oglu25_10_14955	yes	yes	no	yes
On25_10_15001	yes	yes	no	yes
On25_10_15209	yes	yes	no	yes
On25_4_8484	yes	yes	no	yes
Or25_4_13666	yes	yes	no	yes
Or25_4_13729	yes	yes	no	yes
OsJ25_4_17195	yes	yes	no	yes
OsK_chr04_10997	yes	yes	no	yes
OsN_chr04_17195	yes	yes	no	yes
Om25_10_14644	yes	no	yes	yes
OsJ25_10_18835	yes	no	yes	yes
OsN_chr10_18835	yes	no	yes	yes

Supplementary Table S7. Genomic locations of identified *RFLs* as well as conserved non-PPR genes highlighted in the schematic drawing of *RFL* cluster on chromosome 10 in nine *Oryza* species shown in Figure 3.

	Oryza sativa indica			*Oryza sativa japonica*			*Oryza rufipogon*		
	RFL	genomic location	orientation	*RFL*	genomic location	orientation	*RFL*	genomic location	orientation
Identified RFLs (10 or more PPR motifs):	OsI_10_17153	not annotated		OsJ25_10_18827	10: 18,935,634-1	Forward	Or25_10_1	10:16723003-167	Forward
	OsI_10_17163	10: 17,858,774-17,861,422		OsJ25_10_18835	10: 18,946,408-1	Forward	Or25_10_1	10:16850346-168	Forward
	OsI_10_17170	not annotated		OsJ25_10_18966	10: 19,057,033-19,058,658		Or25_10_2	10:16935417-169	Reverse
	OsI_10_17173	10: 17,882,154-17,884,550		OsJ25_10_27808	10: 19,142,225-19,143,607		Or25_10_2	10:16643130-166	Reverse
	OsI_10_17315	not annotated		OsJ25_10_28127	10: 18,847,119-18,848,561		Or25_10_2	10:16606601-166	Reverse
	OsI_10_25889	not annotated		OsJ25_10_28139	10: 18,833,613-18,836,003				
	OsI_10_26225	10: 17,763,649-17,765,082		OsJ25_10_28148	10: 18,823,690-18,825,315				
	OsI_10_26278	not annotated							

	Oryza brachyantha			*Oryza nivara*			*Oryza punctata*		
	RFL	genomic location	orientation	*RFL*	genomic location	orientation	*RFL*	genomic location	orientation
Identified RFLs (10 or more PPR motifs):				On25_10_15001	ONIVA10G16110	Forward	Op25_10_	10:218067	Forward
				On25_10_15209	10:16943550-16945	Forward	Op25_10_	10:220319	Forward
				On25_10_15335	10:17041335-17042	Forward	Op25_10_	10:216816	Reverse
				On25_10_24061	10:17291394-17292	Reverse			
				On25_10_24532	10:16816718-16818	Reverse			
				On25_10_24635	10:16703643-16706	Reverse			

	Oryza glumipatula			*Oryza meridionalis*			*Oryza barthii*		
	RFL	genomic location	orientation	*RFL*	genomic location	orientation	*RFL*	genomic location	orientation
Identified RFLs (10 or more PPR motifs):	Oglu25_10_14955	10:185513	Forward	Om25_10_	10:1404235	for	Oba25_10	10:155369	Forward
	Oglu25_10_22524	10:193529	Reverse	Om25_10_	10:1407519	for	Oba25_10	10:155463	Forward
	Oglu25_10_23193	10:186733	Reverse	Om25_10_	10:1407984	for	Oba25_10	10:156472	Forward
	Oglu25_10_23515	10:183051	Reverse	Om25_10_	10:1419912	for	Oba25_10	10:157291	Reverse
	Oglu25_10_23608	10:181600	Reverse	Om25_10_	10:1431057	rev	Oba25_10	10:154391	Reverse
				Om25_10_	10:1399431	rev	Oba25_10	10:153299	Reverse
				Om25_10_	10:1395785	rev			
				Om25_10_	10:2191809-	rev			

Protein name	letter code in Figure 3	*O. sativa indica* genomic location	gene ID	orientation	*O. sativa japonica* genomic location	gene ID	orientation	*O. rufipogon* genomic location	gene ID	orientation
Alphagalactosidase	a	10:17613650-17	BGIOSGA033189	Forward	10:18739502-18	Os10g0493600	Forward	10:165173	ORUFI10G15150	Forward
KH-domain-containing protein	b	10:17705118-17	BGIOSGA033198,	Forward	10:18815467-18	OS10G0495000,	Forward	10:165987	ORUFI10G15280	Forward
DNA-directed RNA polymerase	c	10:17780154-17	BGIOSGA031619	Forward	10:18858099-18	OS10G0495600	Forward	10:166572	ORUFI10G15380	Forward
Acyltransferase	d	10:17836522-17	BGIOSGA033204	Forward	10:18918078-18	OS10G0497100	Forward	10:167123	not annotated	Forward
Serine/threonine-protein kinase	e	10:17908433-17	BGIOSGA033208,	Forward	10:18971435-18	OS10G0497600	Forward	10:167669	ORUFI10G15540	Forward
Glutamyl-tRNA reductase	f	10:18117298-18	BGIOSGA031606	Reverse	10:19159522-19	Os10g0502400,	Reverse	10:169526	ORUFI10G15830	Reverse

Protein name	letter code in Figure 3	*O. brachyantha* genomic location	gene ID	orientation	*O. nivara* genomic location	gene ID	orientation	*O. punctata* genomic location	gene ID	orientation
Alphagalactosidase	a	10:110678	OB10G21390	Forward	10:16720564-16	ONIVA10G16140	Forward	10:215914	OPUNC10G12740	Forward
KH-domain-containing protein	b	10:111408	OB10G21510	Forward	10:16803226-16	ONIVA10G16260	Forward	10:216774	OPUNC10G12850	Forward
DNA-directed RNA polymerase	c	10:111571	OB10G21550	Forward	10:16830852-16	ONIVA10G16280	Forward	10:217043	OPUNC10G12900	Forward
Acyltransferase	d	10:112083	OB10G21640	Forward	10:16935700-16	ONIVA10G16390	Forward	10:218017	OPUNC10G13010	Forward
Serine/threonine-protein kinase	e	10:112305	OB10G21680	Forward	10:16664689-16	ONIVA10G16100	Reverse	10:218346	OPUNC10G13040	Forward
Glutamyl-tRNA reductase	f	10:114047	OB10G22030	Reverse	10:17308865-17	ONIVA10G16830	Reverse	10:221511	OPUNC10G13390	Reverse

Protein name	letter code in Figure 3	*O. glumipatula* genomic location	gene ID	orientation	*O. meridionalis* genomic location	gene ID	orientation	*O. barthii* genomic location	gene ID	orientation
Alphagalactosidase	a	10:18197618-1819912	OGLUM10G14180	Forward	10:138313	OMERI10G1	Forward	10:153462	OBART10G	Forward
KH-domain-containing protein	b	10:18299900-1830175	OGLUM10G14300	Forward	10:139522	OMERI10G1	Forward	10:154312	OBART10G	Forward
DNA-directed RNA polymerase	c	10:18352960-1835630	OGLUM10G14320	Forward	10:140108	OMERI10G1	Forward	10:154663	OBART10G	Forward
Acyltransferase	d	10:18425053-1842638	OGLUM10G14420	Forward	10:140348	partially OM	Forward	10:155281	OBART10G	Forward
Serine/threonine-protein kinase	e	10:18462301-1846572	OGLUM10G14440	Forward	10:141110	OMERI10G1	Forward	10:155668	OBART10G	Forward
Glutamyl-tRNA reductase	f	10:18690473-1869156	OGLUM10G14740	Forward	10:14329095-14330183		Reverse	10:157443	OBART10G	Reverse

Supplementary Table S8. Results of Z-score test analysis of amino acid combinations at positions 5 and 35 between P-class and RFL proteins.

	ND	NN	SD	SN	TN	NE	TD	GD	GN	NT	RD	NS	NG	DN	GG	GE	SE	DS	RN	AD	SS	CN	LN	GS	EN	DT	GT	AS	NQ	IS
RFLs	489	305	223	173	149	135	129	105	74	65	63	60	42	34	29	28	23	23	22	20	20	18	17	17	16	16	15	14	13	13
P-class	5919	3427	1352	1008	1380	240	2004	382	554	1163	378	1098	263	484	126	118	182	354	91	452	309	63	92	201	86	233	87	163	52	55
Z-score	-0.9513	-2.0331	-10.5845	-9.7706	-3.6667	-21.37	2.3726	-12.099	-4.2952	2.808.	-6	2.8723	-4	0.7	-5	-5	-2	0.9	-5	2.6076	0.9	-5	-3	-0	-3	0.6	-3	-0	-4	-4
significant	not	not	yes	yes	yes	yes	not	yes	yes	yes Pclass	yes	yes Pclass	yes	not	yes	yes	not	not	yes	yes Pclass	not	yes	yes	not	yes	not	yes	not	yes	yes

yes	significantly more frequent in RFLs compared to P-class
not	not more frequent in RFLs compared to P-class
yes Pclass	significantly more frequent in P-class compared to RFLs

Results significant at $p < 0.01$

Supplementary Table S9. Frequency of amino acid combinations at positions 5 and 35 in P-class and RFL proteins.

P-class				RFLs			
amino acid at position 5 and 35	%	RNA base	Sum	amino acid at position 5 and 35	%	RNA base	Sum
ND	17.3	U		ND	18.0	U	
NN	10.0	C		NN	11.2	C	
TD	5.8	G		SD	8.2	G	
TN	4.0	A		SN	6.4	A	
SD	3.9	G		TN	5.5	A	
NT	3.4	C		TD	4.7	G	54.0
NS	3.2	C		NE	5.0		
SN	2.9	A	50.64	GD	3.9		
TT	1.7			GN	2.7		
GN	1.6			NT	2.4		
DN	1.4			RD	2.3		
TS	1.3			NS	2.2		
AD	1.3			NG	1.5		
GD	1.1			DN	1.3		
RD	1.1			GG	1.1		
DS	1.0			GE	1.0		
ST	1.0			SE	0.8		
SS	0.9			DS	0.8		
KD	0.9			RN	0.8		
VD	0.8			AD	0.7		
NG	0.8			SS	0.7		
NE	0.7			CN	0.7		
DT	0.7			LN	0.6		
AN	0.6			GS	0.6		
DD	0.6			EN	0.6		
RS	0.6			DT	0.6		
GS	0.6			GT	0.6		
CD	0.6			AS	0.5		
SE	0.5			NQ	0.5		
SG	0.5			IS	0.5		
